# Validation of Chronic Restraint Stress Model in Young Adult Rats for the Study of Depression Using Longitudinal Multimodal MR Imaging

**DOI:** 10.1523/ENEURO.0113-20.2020

**Published:** 2020-07-29

**Authors:** Bhedita J. Seewoo, Lauren A. Hennessy, Kirk W. Feindel, Sarah J. Etherington, Paul E. Croarkin, Jennifer Rodger

**Affiliations:** 1 Experimental and Regenerative Neurosciences, School of Biological Sciences, The University of Western Australia, 6009 Western Australia, Australia; 2 Brain Plasticity Group, Perron Institute for Neurological and Translational Science, 6009 Western Australia, Australia; 3 Centre for Microscopy, Characterisation & Analysis, Research Infrastructure Centres, The University of Western Australia, 6009 Western Australia, Australia; 4 School of Biomedical Sciences, The University of Western Australia, 6009 Western Australia, Australia; 5 College of Science, Health, Engineering and Education, Murdoch University, Perth, 6150 Western Australia, Australia; 6 Mayo Clinic Depression Center, Department of Psychiatry and Psychology, Mayo Clinic, Rochester, MN 55905

**Keywords:** animal model, chronic restraint stress, depression, hippocampus, resting-state fMRI, spectroscopy

## Abstract

Prior research suggests that the neurobiological underpinnings of depression include aberrant brain functional connectivity, neurometabolite levels, and hippocampal volume. Chronic restraint stress (CRS) depression model in rats has been shown to elicit behavioral, gene expression, protein, functional connectivity, and hippocampal volume changes similar to those in human depression. However, no study to date has examined the association between behavioral changes and brain changes within the same animals. This study specifically addressed the correlation between the outcomes of behavioral tests and multiple 9.4 T magnetic resonance imaging (MRI) modalities in the CRS model using data collected longitudinally in the same animals. CRS involved placing young adult male Sprague Dawley rats in individual transparent tubes for 2.5 h daily over 13 d. Elevated plus maze (EPM) and forced swim tests (FSTs) confirmed the presence of anxiety-like and depression-like behaviors, respectively, postrestraint. Resting-state functional MRI (rs-fMRI) data revealed hypoconnectivity within the salience and interoceptive networks and hyperconnectivity of several brain regions to the cingulate cortex. Proton magnetic resonance spectroscopy revealed decreased sensorimotor cortical glutamate (Glu), glutamine (Gln), and combined Glu-Gln (Glx) levels. Volumetric analysis of T2-weighted images revealed decreased hippocampal volume. Importantly, these changes parallel those found in human depression, suggesting that the CRS rodent model has utility for translational studies and novel intervention development for depression.

## Significance Statement

Peripheral biomarker studies suggest that chronic restraint stress (CRS) is a valid rodent model of depression, with animals exhibiting similar gene expression and protein changes, aberrant brain functional connectivity, and reduced hippocampal volume found in human depression. However, the present study is the first to demonstrate hyperconnectivity and hypoconnectivity, hippocampal atrophy, and decreased sensorimotor cortical glutamate (Glu) and glutamine (Gln) levels in the same young adult male rats postrestraint and the correlation of these measures with changes in behavior. Importantly, these changes are similar to anomalies found in humans with depression, which also correlate with patient symptoms. Present findings reinforce the usefulness of the CRS model for translational studies, intervention development, and multimodal molecular and imaging studies.

## Introduction

Major depressive disorder is a debilitating neuropsychiatric disease with significant morbidity and mortality. The diagnosis of depression in humans is based on persistent negative mood, clinical symptoms, and behavioral changes. However, diagnosing depression based solely on clinical features leads to suboptimal outcomes in research studies and clinical practice. Considerable effort has been focused on addressing the biological heterogeneity of depression with biomarkers, including the use of magnetic resonance imaging (MRI) techniques. For example, the cingulate cortex, a critical node of the default mode network (DMN), is one of the most extensively investigated brain regions in the context of mood disorders in MRI studies because of its role in the modulation of emotional behavior (Drevets et al., 2008; Davey et al., 2012; Rolls et al., 2019). Resting-state functional MRI (rs-fMRI) studies of depression demonstrate functional connectivity changes in the DMN, along with other resting-state networks (RSNs) such as the salience network and the interoceptive network which are involved in processing emotions and sensory stimuli and regulating the internal state (Paulus and Stein, 2010; Mulders et al., 2015). These alterations in functional connectivity within RSNs are associated with neurometabolite [e.g., glutamine (Gln); glutamate (Glu); GABA] imbalances in depression, measured non-invasively using proton magnetic resonance spectroscopy (^1^H-MRS; Lener et al., 2017). Additionally, human MRI studies reproducibly detect reduced hippocampal volumes in patients with depression compared with age-matched healthy controls (Videbech and Ravnkilde, 2004).

Although significant progress has been made in understanding the mechanisms underpinning major depressive disorder, the causality of neuroimaging findings is difficult to infer. For example, the state versus trait nature of human imaging findings are often uncertain and difficult to study (Sheline, 2011; Brown et al., 2014). In contrast, temporal relationships between biological findings and depression-like behaviors can be studied in animal models. Moreover, MRI-based techniques can be used to investigate the same biological changes in humans and animals, allowing direct comparison of validated outcome measures. Furthermore, combining repeated behavioral and MRI measures within the same animals allows the exploration of correlation between those measures. Therefore, animal models are an indispensable tool for studying etiology, progress, and treatment of depression.

Chronic restraint stress (CRS) in Sprague Dawley rats has been shown to elicit behavioral, gene expression, protein, brain functional connectivity, and hippocampal volume changes similar to those in patients with depression. However, no study to date has examined the association between multimodal MRI measures and behavioral changes within the same animals in the CRS model of depression (Lee et al., 2009; Henckens et al., 2015; Wang et al., 2017). This model involves restraining animals’ movements for at least 2 h/d for several days (Wang et al., 2017); the continuous and predictable stress is designed to mimic everyday human stress, such as daily repetition of a stressful job and familial stresses. Our study aimed to bring previous MRI findings in CRS animals together and investigate the relationship between neurobiological and behavioral changes in the CRS rat model by performing multimodal MRI (rs-fMRI, ^1^H-MRS, and structural MRI) and behavioral tests on the same young adult male rats before and after induction of the model.

## Materials and Methods

### Ethics statement

Experimental procedures were approved by The University of Western Australia (UWA) Animal Ethics Committee (RA/3/100/1640) and conducted in accordance with the National Health and Medical Research Council Australian code for the care and use of animals for scientific purposes. All investigators were trained in animal handling by the UWA Programme in Animal Welfare, Ethics, and Science (PAWES) and had valid Permission to Use Animals (PUA) licenses.

### Animals

Young adult male Sprague Dawley rats (*n* = 33; 150–200 g; six to seven weeks old) from the local Animal Resources Centre were housed in pair under temperature-controlled conditions on a 12/12 h light/dark cycle. Food and water were freely provided, except during the CRS procedure and fasting before the sucrose preference test (SPT). All rats acclimatized to their new environment for one week following their arrival. Behavioral tests and MRI scans were conducted at baseline and after the final restraint procedure. A control group of (*n* = 8) animals underwent all procedures except CRS.

### CRS procedure

The CRS procedure was conducted on a bench located on the opposite side of the large animal holding room facing the wall. Each session was conducted between 12:30 and 3:30 P.M. in effort to avoid effects associated with the circadian rhythm. In brief, rats were weighed and placed in a transparent tube (size of the tube depending on weight of animal, see Table 1) for 2.5 h/d for 13 consecutive days as performed on Sprague Dawley rats in previous studies (Bravo et al., 2009; Ulloa et al., 2010; Stepanichev et al., 2014). The length of each restraint was adjusted to limit limb movements using tail gates. Following CRS, rats were returned to their home cages. Healthy control animals were not restrained and remained in their home cages.

**Table 1:** Weight of animals and size of restraints

Body weightof animal (g)	Diameter ofrestraint (cm)	Maximum lengthof restraint (cm)
<255	5	19
255–300	5	23
>300	6	21

### Behavioral tests

**Figure 1.**
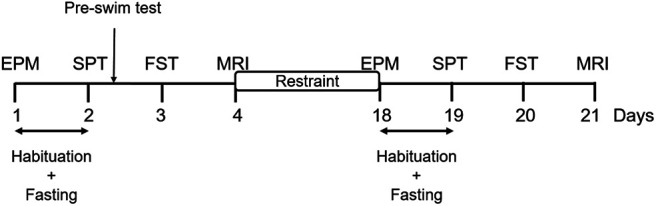
Experimental timeline. The experiment consisted of an initial one-week period of habituation on arrival of the animals, after which the rats underwent EPM test (day 1). Following EPM, animals were habituated to single housing and sucrose solution for 8 h and deprived of food and water overnight (days 1–2). SPT was conducted the next day (day 2), followed by a preswim test. FST was conducted on day 3 and MRI on day 4. The animals then underwent CRS for 2.5 h daily for 13 consecutive days. The day after the end of CRS, animals underwent behavioral tests (days 18–20) and MRI (day 21) in the same order without a preswim test.

Elevated plus maze (EPM) and forced swim tests (FSTs) were used to confirm increased anxiety-related behaviors and learned-helplessness induced by the CRS paradigm in rats as previously described in several preclinical studies (Suvrathan et al., 2010; Ulloa et al., 2010; Chiba et al., 2012; Bogdanova et al., 2013). Behavioral tests were conducted over a period of 3 d (Fig. 1). On the first day, the animals were subjected to EPM test (Walf and Frye, 2007). After EPM, the animals were habituated to single housing and 1% sucrose solution as described below and deprived of food and water overnight. The next day, the SPT (Willner et al., 1987) was conducted and on the third day, the animals underwent the FST (Slattery and Cryan, 2012). All behavioral testing occurred between 8:30 and 11 A.M. The full behavioral dataset can be obtained from the corresponding author on request.

### EPM

EPM was conducted as detailed in Walf and Frye (2007). The apparatus consisted of two open arms (without walls or railing) and two closed arms, crossed in the middle perpendicularly to each other, and a center area (10 × 10 cm). Each arm was 50 cm long and 10 cm wide, and the enclosed arms had 40-cm high walls. Each arm of the maze was attached to sturdy legs, such that it was elevated 60 cm off the floor. The maze was placed in a way to ensure similar levels of illumination on both open and closed arms. One animal was tested at a time and after each trial, all arms and the center area were wiped with 70% ethanol to remove olfactory cues. The animal was placed in the center of the maze facing the same open arm, away from the experimenter. The animal was allowed to move freely in the maze for 5 min and the whole procedure was video recorded from ∼120 cm above the platform using a GoPro HERO7. The experimenters stayed in the room during the procedure, but unnecessary movements and noise were minimized.

EPM data were analyzed manually by researchers blinded to experimental group and timepoint. The number of entries and time spent in closed and open arms were measured. Additionally, the number and duration of rearing and grooming were also measured to investigate anxiety-related behaviors (Walf and Frye, 2007). The number of entries and time spent in the center of the maze and behaviors such as head shaking, head dips and stretching were not considered. One animal fell off the open arm during baseline testing and was re-placed onto the maze to continue the whole 5-min testing, but the data were excluded from the analyses (Walf and Frye, 2007).

### SPT

Immediately following EPM, animals were habituated to single housing and to sucrose solution (Fig. 1). Animals were placed in individual cages with *ad libitum* access to food pellets and two 600-ml bottles, one bottle containing fresh 1% sucrose solution [D-(+)-Sucrose, AnalaR NORMAPUR analytical reagent, VWR International BVBA] and the other containing tap water. The animals were trained to this condition for 8 h. Rats were given a free choice between the two bottles and the position of the bottles was switched 4 h after the start of single housing to prevent side preference in drinking behavior. Overnight food and water deprivation was applied at the end of the 8-h habituation for up to 16 h.

The next day, SPT was performed according to a previous study with some modifications (Willner et al., 1987). Water and sucrose solution bottles were weighed, labeled, and placed in corresponding cages. The position of the bottles was switched 30 min after the start of the SPT. Thirty minutes later, the bottles were removed and re-weighed, and the animals were re-housed in their original cages. Sucrose preference was calculated as a percentage of the total amount of liquid ingested (sucrose preference = sucrose consumption (g)/[sucrose consumption (g) + water consumption (g)]).

### FST

FST was conducted as detailed in Slattery and Cryan (2012). Briefly, 20-l white opaque plastic buckets (41 cm high, 28 cm wide) were filled up to a depth of 30 cm with water at 23–25°C. At this depth, the rats could not touch the bottom of the bucket with their tails or hind limbs. Up to four buckets were used at a time, and the buckets were emptied, cleaned, and refilled between animals. At baseline, 24 h before the FST session (on SPT day), rats were exposed to a preswim test for 10 min by placing them in the water-filled buckets (Slattery and Cryan, 2012). The next day, and at the end of the restraint period, rats underwent 6 min of FST (Fig. 1) and the procedure was video recorded from ∼50 cm above the buckets using a GoPro HERO7.

FST data were analyzed manually by researchers blinded to experimental group and timepoint using a time-sampling technique (Slattery and Cryan, 2012). The first 5 min of the video recording was split into 5-s intervals, and the predominant behavior in each 5-s period was rated. The following escape behaviors were scored: (1) swimming, with horizontal movements throughout the bucket including crossing into another quadrant and diving; (2) climbing, with upward movements of the forepaws along the side of bucket; (3) immobility, with minimal movements necessary to keep their head above water; and (4) latency, defined as the time taken to exhibit the first immobility behavior. Grooming, head shaking and number of fecal boli were not considered. Trials during which the animals managed to escape more than once or were floating horizontally for the duration of the test (with most of their body being completely dry at the end) were excluded from the analyses.

### MRI data acquisition

#### Animal preparation

MRI data were acquired the day after the FST. Because of the well-documented effect of anesthesia on RSNs, a combined medetomidine-isoflurane anesthetic protocol was chosen because it produces similar RSN connectivity as in the awake condition (Paasonen et al., 2018), maintains strong intercortical and cortical-subcortical connectivity (Grandjean et al., 2014; Bukhari et al., 2017) and provides stable sedation for over 4 h and reproducible data from repeated fMRI experiments on the same animal one week apart (Lu et al., 2012). The rat was preanaesthetized using isoflurane (Isothesia, Henry Schein Medical Animal Health) in an induction chamber (4% isoflurane in medical air, 2 l/min). Once fully anaesthetized, the animal was transferred to a heated imaging cradle and anesthesia was maintained with a nose cone (2% isoflurane in medical air, 1 l/min). Body temperature and respiratory rate were monitored using a PC-SAM Small Animal Monitor (SA Instruments Inc., 1030 System). An MR-compatible computer feedback heating blanket was used for maintaining animal body temperature at 37°C (± 0.5°C). A 25G butterfly catheter (SV*25BLK, Terumo Australia Pty Ltd) was implanted subcutaneously in the left flank of the animal to deliver a 0.05–0.1 mg/kg bolus injection and continuous infusion of medetomidine (1 mg/ml, Ilium Medetomidine Injection, Troy Laboratories Pty. Limited) at 0.15 mg/kg/h using a single syringe infusion pump (Legato 100 Syringe Pump, KD Scientific Inc.). Once the animal’s breathing rate dropped to 50 breaths/min, isoflurane was gradually reduced to 0.5–0.75%. These anesthetic doses were empirically determined to ensure the respiratory rate of the animals was between 50 and 80 breaths/min. rs-fMRI scans were started only after the isoflurane concentration had been reduced for at least 15 min, and the physiology of the animal was stable during that time. After the MRI procedure, medetomidine was antagonized by an injection of 0.1 mg/kg atipamezole (5.0 mg/ml, Ilium Atipamezole Injection, Troy Laboratories Pty. Limited) using a 29-G insulin syringe (BD Ultra-Fine Insulin Syringe, Becton Dickinson Pty Ltd).

#### MRI acquisition parameters

**Figure 2.**
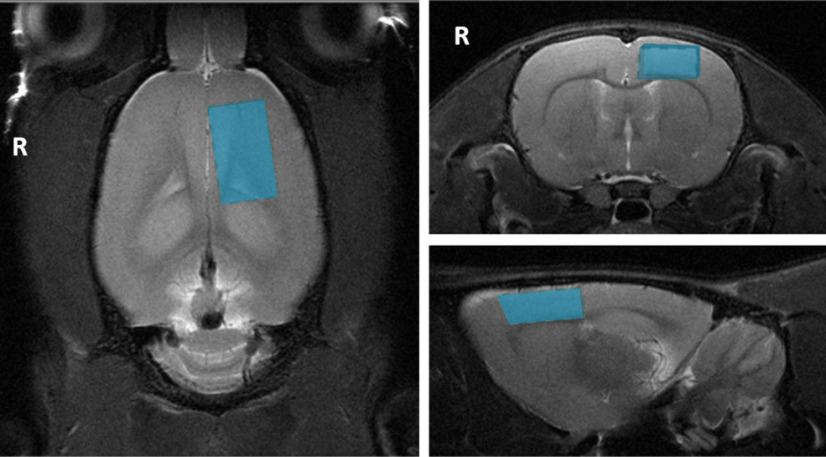
Voxel position for proton magnetic resonance spectroscopy. The figure shows the position of the voxel of interest (size of 3.5 × 2 × 6 mm) on the left sensorimotor cortex on T2-weighted images for proton magnetic resonance spectroscopy. R denotes right hemisphere.

All MR images were acquired with a Bruker Biospec 94/30 small animal MRI system operating at 9.4 T (400 MHz, H-1), with an Avance III HD console, BGA-12SHP imaging gradients, a 72-mm (inner diameter) volume transmit coil and a rat brain surface quadrature receive coil using the imaging protocol as detailed in Seewoo et al. (2018, 2019). The acquisition protocol included the following sequences: (1) multislice 2D RARE (rapid acquisition with relaxation enhancement) sequence for three T2-weighted anatomic scans (TR = 2500 ms, TE = 33 ms, matrix = 280 × 280, pixel size = 0.1 × 0.1 mm^2^, 21 coronal and axial slices, 20 sagittal slices, thickness = 1 mm); (2) single-shot gradient-echo echoplanar imaging (TR = 1500 ms, TE = 11 ms, matrix = 94 × 70, pixel size = 0.3 × 0.3 mm^2^, 21 coronal slices, thickness = 1 mm, flip angle = 90°, 300 volumes, automatic ghost correction order = 1, receiver bandwidth = 300 kHz) for resting-state; and (3) point-resolved spectroscopy (PRESS) sequence with one 90° and two 180° pulses and water suppression for ^1^H-MRS (TE = 16 ms, TR = 2500 ms) with 64 averages with a 3.5 × 2 × 6 mm^3^ voxel placed over the left sensorimotor cortex (Fig. 2). The sensorimotor cortex was chosen because: (1) it is a key brain region involved in psychomotor retardation (Zarrinpar et al., 2006; Pineda, 2008), an important but poorly understood clinical feature of depression (Buyukdura et al., 2011; Ilamkar, 2014); (2) brain stimulation has been reported to decrease the severity of psychomotor retardation in patients with depression (Buyukdura et al., 2011) and increase Glu and Gln levels in the sensorimotor cortex of healthy Sprague Dawley rats (Seewoo et al., 2019); (3) rostral regions such as the sensorimotor cortex have higher signal-to-noise ratio (SNR) when using a surface coil (Xu et al., 2013b); and (4) larger voxel sizes can be used without white matter contamination, leading to higher SNR and greater reliability and reproducibility of measured metabolite concentrations. ^1^H-MRS data were acquired from the left cortex only to facilitate comparisons with human depression studies, which mostly examine neurometabolite changes in the left hemisphere (Moriguchi et al., 2019).

### Analysis steps

#### Behavioral data

EPM and FST videos were scored blind by two trained observers to establish the most reliable measures. Based on their low interindividual scorer variability (<12%), exploration (into and within closed and open arms separately), grooming and rearing for EPM, and swimming and climbing (separately and combined as “total activity”), immobility, and latency to first immobility behavior for FST were selected for statistical analysis. Sucrose preference (%) was calculated as sucrose consumption (g)/[sucrose consumption (g) + water consumption (g)].

#### rs-fMRI data

To maximize the use of collected data, rs-fMRI data and T2-weighted images collected using the same acquisition and anesthesia protocols from a previous study (Seewoo et al., 2019) in adult (six to eight weeks old, 150–250 g) male Sprague Dawley rats were also included in the analyses for the baseline timepoint (Table 2). These animals did not undergo any behavioral testing or intervention before the acquisition of MRI data. All rs-fMRI data were preprocessed and analyzed in the same way. Preprocessing of data included: (1) export into DICOM format from ParaVision 6.0.1 (Bidgood et al., 1997); (2) conversion into NifTI using the dcm2niix converter (64-bit Linux version May 5, 2016; Rorden et al., 2007); (3) reorienting the brain into left-anterior-superior (LAS) axes (radiologic view); and (4) skull-stripping using the qimask utility from QUIT (QUantitative Imaging Tools; Wood, 2018). The voxel sizes were then upscaled by a factor of 10 (Tambalo et al., 2015).

**Table 2:** Statistical table indicating the results of all analyses

No	Fig.	Description^*^		Statistics	Power
EPM test					
aa	3*A*	Open arm #	CRS group (*n* = 24/timepoint)	Paired median difference= –1.0 *p* = 0.0	–1.0, –1.0 baseline = 0.88 ± 0.18 restraint = 0.32 ± 0.13
ab			Healthy group (*n* = 8/timepoint)	Paired median difference= –1.0 *p* = 0.246	–1.0, –1.0 baseline = 1.75 ± 0.62 restraint = 0.38 ± 0.26
ac		Open arm time	CRS group (*n* = 24/timepoint)	Paired median difference= –1.5 *p* = 0.15	–8.0, 0.0 baseline = 5.79 ± 1.43 s restraint = 2.52 ± 1.15 s
ad			Healthy group (*n* = 6/timepoint)	Paired median difference= –3.0 *p* = 0.38	–24.0, 0.0 baseline = 6.83 ± 3.89 s restraint = 2.63 ± 2.01 s
ae	3*B*	Closed arm #	CRS group (*n* = 24/timepoint)	Paired Cohen’s *d* = 0.462 *p* = 0.0282	–0.0122, 0.856 baseline = 5.63 ± 0.66 restraint = 7.20 ± 0.74
af			Healthy group (*n* = 8/timepoint)	Paired Cohen’s *d* = 0.114 *p* = 0.639	–0.647, 0.51 baseline = 8.00 ± 1.72 restraint = 8.63 ± 2.13
ag		Closed arm time	CRS group (*n* = 24/timepoint)	Paired Cohen’s *d* = 0.212 *p* = 0.47	–0.415, 0.777 baseline = 220.83 ± 9.06 s restraint = 230.60 ± 7.81 s
ah			Healthy group (*n* = 8/timepoint)	Paired Cohen’s *d* = 0.056 *p* = 0.888	–0.848, 0.851 baseline = 221.75 ± 12.82 s restraint = 224.13 ± 16.89 s
ai	3*C*	Grooming #	CRS group (*n* = 24/timepoint)	Paired Cohen’s *d* = –0.921 *p* = 0.001	–1.41, –0.357 baseline = 4.63 ± 0.64 restraint = 2.28 ± 0.31
aj			Healthy group (*n* = 8/timepoint)	Paired Cohen’s *d* = 0.232 *p* = 0.464	–0.511, 1.17 baseline = 3.00 ± 1.10 restraint = 3.63 ± 0.78
ak		Grooming time	CRS group (*n* = 23/timepoint)	Paired Cohen’s *d* = –0.642 *p* = 0.0016	–1.09, –0.185 baseline = 24.70 ± 3.93 s restraint = 14.48 ± 3.30 s
al			Healthy group (*n* = 8/timepoint)	Paired Cohen’s *d* = –0.326 *p* = 0.411	–1.11, 0.352 baseline = 24.63 ± 9.89 s restraint = 17.75 ± 3.70 s
am	3*D*	Rearing #	CRS group (*n* = 23/timepoint)	Paired Cohen’s *d* = –0.2 *p* = 0.422	–0.732, 0.327 baseline = 17.79 ± 0.86 restraint = 16.92 ± 1.14
an			Healthy group (*n* = 7/timepoint)	Paired Cohen’s *d* = 0.533 *p* = 0.19	–0.852, 1.13 baseline = 16.14 ± 1.52 restraint = 17.38 ± 1.82
ao		Rearing time	CRS group (*n* = 24/timepoint)	Paired Cohen’s *d* = 0.187 *p* = 0.501	–0.364, 0.73 baseline = 41.38 ± 1.72 s restraint = 44.36 ± 3.36 s
ap			Healthy group (*n* = 8/timepoint)	Paired Cohen’s *d* = 0.117 *p* = 0.618	–0.639, 0.552 baseline = 41.88 ± 5.43 s restraint = 43.38 ± 3.42 s
SPT					
aq		CRS group (*n* = 25/timepoint)		Paired Cohen’s *d* = 0.289 *p =* 0.312	–0.294, 0.889 baseline = 0.63 ± 0.03 restraint = 0.69 ± 0.04
ar		Healthy group (*n* = 5/timepoint)		Paired Cohen’s *d* = –0.191 *p* = 0.807	–1.52, 0.831 baseline = 0.81 ± 0.03 restraint = 0.78 ± 0.07
FST					
as	4*A*	Total activity	CRS group (*n* = 19/timepoint)	Paired Cohen’s *d* = –0.62 *p* = 0.0414	–1.19, –0.0902 baseline = 42 ± 2 restraint = 37 ± 1
at			Healthy group (*n* = 8/timepoint)	Paired Cohen’s *d* = –0.726 *p* = 0.22	–1.91, 0.489 baseline = 45 ± 3 restraint = 40 ± 2
au	4*B*	Swimming	CRS group (*n* = 19/timepoint)	Paired Cohen’s *d* = 0.0269 *p* = 0.896	–0.526, 0.489 baseline = 26 ± 2 restraint = 26 ± 2
av			Healthy group (*n* = 8/timepoint)	Paired Cohen’s *d* = 0.0 *p* = 0.969	–1.54, 1.17 baseline = 30 ± 3 restraint = 30 ± 4
aw		Climbing	CRS group (*n* = 19/timepoint)	Paired Cohen’s *d* = –0.696 *p* = 0.021	–1.22, –0.202 baseline = 16 ± 2 restraint = 11 ± 1
ax			Healthy group (*n* = 8/timepoint)	Paired Cohen’s *d* = –0.8 *p* = 0.12	–1.92, 0.327 baseline = 15 ± 2 restraint = 10 ± 2
ay	4*C*	Immobility	CRS group (*n* = 19/timepoint)	Paired Cohen’s *d* = 0.611 *p* = 0.0438	0.0902, 1.19 baseline = 18 ± 2 restraint = 23 ± 1
az			Healthy group (*n* = 8/timepoint)	Paired Cohen’s *d* = 0.726 *p* = 0.22	0.0902, 1.19 baseline = 15 ± 3 restraint = 20 ± 2
ba		Latency	CRS group (*n* = 19/timepoint)	Paired Cohen’s *d* = –1.34 *p* = 0.0006	–2.03, –0.578 baseline = 119 ± 9 s restraint = 71 ± 7 s
bb			Healthy group (*n* = 8/timepoint)	Paired Cohen’s *d* = –0.0967 *p* = 0.92	–1.59, 1.21 baseline = 101 ± 32 restraint = 95 ± 16
rs-fMRI					
bc	5*A*,*B*	ICA/dual regression	CRS group with all restraint data [baseline: *n* = 11 from current cohort and *n* = 22from Seewoo et al. (2019); restraint: *n* = 15]	Dual regression *p* < 0.05	
bd			CRS group with restraint data based on FST findings [baseline: *n* = 11 from currentcohort and *n* = 22 from Seewoo et al. (2019); restraint: *n* = 9]	Dual regression *p* < 0.05	
be	5*C*		Salience network CRS group (baseline: *n* = 9 from current cohort; restraint: *n* = 15)	Unpaired Cohen’s *d* = –2.33 *p* = 0.0	–3.2, –1.29 baseline = 36 ± 3 restraint = 19 ± 1
bf			Salience network Healthy group (baseline: *n* = 5 from current cohort; restraint: *n* = 8)	Unpaired Cohen’s *d* = –0.208 *p* = 0.718	–1.68, 0.93 baseline = 29 ± 5 restraint = 27 ± 4
bg	5*D*		Interoceptive network CRS group (baseline: *n* = 9 from current cohort; restraint: *n* = 15)	Unpaired Cohen’s *d* = –1.38 *p* = 0.0032	–2.13, –0.574 baseline = 39 ± 5 restraint = 23 ± 2
bh			Interoceptive network Healthy group (baseline: *n* = 5 from current cohort; restraint: *n* = 8)	Unpaired Cohen’s *d* = –0.539 *p* = 0.374	–1.47, 0.685 baseline = 27 ± 2 restraint = 22 ± 4
bi	6*A*	Seed-based analysis	CRS group with all restraint data [baseline: *n* = 11 from current cohort and *n* = 22from Seewoo et al. (2019); restraint: *n* = 15]	Higher-level FEAT *p* < 0.05, *z* > 2	
bj	6*B*		CRS group (baseline: *n* = 9 from current cohort; restraint: *n* = 15)	Unpaired Cohen’s *d* = 1.51 *p* = 0.0018	0.712, 2.18 baseline = 0.13 ± 0.02 restraint = 0.29 ± 0.03
bk			Healthy group (baseline: *n* = 5 from current cohort; restraint: *n* = 8)	Unpaired Cohen’s *d* = 0.752 *p* = 0.214	–0.318, 1.74 baseline = 0.15 ± 0.03 restraint = 0.24 ± 0.05
Proton magnetic resonance spectroscopy					
bl	7*C*	Gln	CRS group (*n* = 17/timepoint)	Paired Cohen’s *d* = –0.538 *p* = 0.027	–1.19, –0.0217 baseline: 0.53 ± 0.01 restraint: 0.50 ± 0.01
bm			Healthy group (*n* = 8/timepoint)	Paired Cohen’s *d* = 0.743 *p* = 0.174	–0.127, 1.95 baseline = 0.50 ± 0.02 restraint = 0.54 ± 0.02
bn	7*D*	Glu	CRS group (*n* = 17/timepoint)	Paired Cohen’s *d* = –0.711 *p* = 0.0706	–1.46, 0.134 baseline: 1.41 ± 0.02 restraint: 1.35 ± 0.02
bo			Healthy group (*n* = 6/timepoint)	Paired Cohen’s *d* = –1.1 *p* = 0.121	–2.15, –0.122 baseline = 1.48 ± 0.04 restraint = 1.37 ± 0.04
bp	7*E*	Gln + Glu (Glx)	CRS group (*n* = 17/timepoint)	Paired Cohen’s *d* = –0.84 *p* = 0.0186	–1.49, –0.115 baseline: 1.94 ± 0.02 restraint: 1.85 ± 0.03
bq			Healthy group (*n* = 7/timepoint)	Paired Cohen’s *d* = –0.41 *p* = 0.529	–1.83, 1.12 baseline = 1.97 ± 0.04 restraint = 1.92 ± 0.05
br		GABA	CRS group (*n* = 16/timepoint)	Paired Cohen’s *d* = –0.137 *p* = 0.74	–0.959, 0.632 baseline: 0.35 ± 0.01 restraint: 0.34 ± 0.01
bs			Healthy group (*n* = 8/timepoint)	Paired Cohen’s *d* = –0.558 *p* = 0.378	–1.82, 0.926 baseline = 0.37 ± 0.02 restraint = 0.34 ± 0.02
bt		Gln/Glu	CRS group (*n* = 17/timepoint)	Paired Cohen’s *d* = –0.171 *p* = 0.552	–0.767, 0.275 baseline = 0.38 ± 0.01 restraint = 0.37 ± 0.01
bu			Healthy group (*n* = 6/timepoint)	Paired Cohen’s *d* = 1.15 *p* = 0.0632	0.45, 2.51 baseline = 0.35 ± 0.02 restraint = 0.41 ± 0.02
Animal weight and whole-brain volume					
bv	8*A*	Spearman’s rank correlation rho because baseline weights were not normally distributed [*n* = 33 fromcurrent cohort, *n* = 41 from Seewoo et al. (2019) and our unpublished data]		*S* = 28632 *p* = 7.909e-08 *r* = 0.58	Mean whole-brain volume to weight ratio = 6.64 ± 0.16 mm^3^/g
Hippocampal volume					
bw	8*B*	CRS group (*n* = 18/timepoint)		Paired Cohen’s *d* = –0.811 *p* = 0.0032	–1.33, –0.318 baseline: 5.88 ± 0.01 restraint: 5.85 ± 0.01
bx		Healthy group (*n* = 8/timepoint)		Paired Cohen’s *d* = –0.409 *p* = 0.327	–1.64, 0.467 baseline = 5.87 ± 0.01 restraint = 5.85 ± 0.02
Correlations					
by	9*A*	Latency and salience network functional connectivity (not normal; Spearman’s rank correlationrho; *n* = 35)		*R* = 0.180 *S* = 5853.7 *p* = 0.3004 p_adj_ = 0.4440	
bz	9*B*	Latency and interoceptive network functional connectivity (not normal; Spearman’s rank correlationrho; *n* = 35)		*R* = 0.312 *S* = 4909.9 *p* = 0.0678 *p*_adj_ = 0.3627	
ca	9*C*	Salience and interoceptive network functional connectivity (not normal; Spearman’s rank correlationrho; *n* = 37)		*R* = 0.595 *S* = 3414 *p* = 0.0001 *p*_adj_ = 0.0017	
cb	9*D*	Latency and cingulate cortex functional connectivity (not normal; Spearman’s rank correlationrho; *n* = 35)		*R* = –0.484 *S* = 10597 *p* = 0.0032 *p*_adj_ = 0.0320	
cc	9*E*	Cingulate cortex and salience network functional connectivity (not normal; Spearman’s rankcorrelation rho; *n* = 37)		*R* = –0.560 *S* = 13158 *p* = 0.0004 *p*_adj_ = 0.0044	
cd	9*F*	Cingulate cortex and interoceptive network functional connectivity (not normal; Spearman’srank correlation rho; *n* = 37)		*R* = –0.402 *S* = 11828 *p* = 0.0142 *p*_adj_ = 0.1137	
ce	9*G*	Latency and Glx/tCr (latency not normal; Spearman’s rank correlation rho; *n* = 51)		*R* = 0.180 *S* = 18116 *p* = 0.2055 *p*_adj_ = 0.4440	
cf	9*H*	Latency and hippocampal volume (latency not normal; Spearman’s rank correlation rho; *n* = 52)		*R* = 0.262 *S* = 17285 *p* = 0.0605 *p*_adj_ = 0.3627	
cg	9*I*	Postrestraint latency and baseline hippocampal volume of CRS group (normal; Pearson’s product-moment correlation; *n* = 23)		*R* = 0.311 *t*_(21)_ = 1.50 *p* = 0.1480 *p*_adj_ = 0.4440	–0.116, 0.641
ch	9*J*	Hippocampal volume and salience network functional connectivity (salience network functionalconnectivity not normal; Spearman’s rank correlation rho; *n* = 36)		*R* = 0.341 *S* = 5124 *p* = 0.0427 *p*_adj_ = 0.2990	
ci	9*K*	Hippocampal volume and interoceptive network functional connectivity (interoceptive networkfunctional connectivity not normal; Spearman’s rank correlation rho; *n* = 36)		*R* = 0.299 *S* = 5444 *p* = 0.0764 *p*_adj_ = 0.3627	
cj	9*L*	Hippocampal volume and cingulate cortex functional connectivity (cingulate cortex functionalconnectivity not normal; Spearman’s rank correlation rho; *n* = 36)		*R* = –0.431 *S* = 11122 *p* = 0.0091 *p*_adj_ = 0.0822	

Each analysis includes a letter indicator linking the test in the table to the analysis in the text. The link to the corresponding figure, if any, is indicated under Fig. The estimation statistics, critical value, degrees of freedom, and exact *p* values are listed for each test under statistics, and the CIs of the tests and mean ± SE are under power.

* Number of animals are different between groups and among tests because (1) one animal fell off the open arm during baseline EPM testing and baseline and postrestraint EPM data from this animal was excluded from the analyses; (2) FST trials during which the animals managed to escape more than once or were floating horizontally for the duration of the test (with most of their body being completely dry at the end) were excluded from the analyses; (3) sessions during which the CRLB of a metabolite of interest was greater than 20% in the ^1^H-MRS data were excluded from the analyses; (4) not all animals were imaged at baseline and following restraint because of limited access to the MRI instrument and time taken to scan each animal (∼1.5 h per animal); and (5) animals with variable physiology (e.g., rapidly increasing/decreasing breathing rates) during rs-fMRI scans were excluded from the analyses.

All further preprocessing and analyses were performed using FSL v5.0.10 [Functional MRI of the Brain (FMRIB) Software Library; Jenkinson et al., 2012] using the methods described in Seewoo et al. (2020). Single-session independent component analysis (ICA) as implemented in FSL/MELODIC (Multivariate Exploratory Linear Decomposition into Independent Components; Beckmann et al., 2005) was used to de-noise the data (detailed in Seewoo et al., 2018). The de-noised rs-fMRI images were then co-registered to their respective T2-weighted coronal images using six-parameter rigid body registration using FSL/FLIRT (Linear Image Registration Tool; Jenkinson and Smith, 2001; Jenkinson et al., 2002) and normalized to a Sprague Dawley brain atlas (Papp et al., 2014; Kjonigsen et al., 2015; Sergejeva et al., 2015) with nine degrees of freedom “traditional” registration. The atlas was first down-sampled by a factor of eight to better match the voxel size of the 4D functional data. All subsequent analyses were conducted in the atlas standard space.

Multisubject temporal concatenation group-ICA and FSL dual regression analysis were used to determine group differences (baseline, *n* = 33; restraint, *n* = 15), controlling for family-wise error (FWE) and using a threshold-free cluster enhanced (TFCE) technique to control for multiple comparisons. The resulting statistical maps were thresholded to *p* < 0.05.

To investigate the correlation between strong depression-like behaviors on functional connectivity, dual regression was also conducted using a subset of animals in the restraint group. FST measures (immobility, swimming and climbing scores, and latency time) were extracted for the 15 animals scanned following restraint and sorted in order of greatest change in each FST measure. Animals were scored according to their position on the list (1–15). Nine of 15 animals had consistently high scores and were used in the analysis as they exhibited the greatest change in overall behavioral outcomes in FST.

For seed-based analysis, the atlas mask for cingulate cortex was transformed to each individual animal’s functional space. The region of interest (ROI) masks (within the individual functional space) were used to extract the time course from the ICA de-noised data. The time courses were used in a first-level FSL/FEAT (FMRI Expert Analysis Tool version 6.00) analysis to generate a whole-brain correlation map. Higher-level analysis was conducted using ordinary least squares (OLS) simple mixed-effects (Beckmann et al., 2003; Woolrich et al., 2004; Woolrich, 2008) in atlas space (baseline, *n* = 33; restraint, *n* = 15). *Z* (Gaussianized T/F) statistic images were thresholded non-parametrically using clusters determined by *Z* > 2 and a (corrected) cluster significance threshold of *p *=* *0.05 (Worsley, 2001).

#### ^1^H-MRS

^1^H-MRS data were analyzed in LCModel (Linear Combination of Model spectra, version 6.3-1L; Provencher, 2001) using a set of simulated basis set provided by the software vendor. For data quality control, the linewidth (full width at half-maximum; FWHM) for each scan was calculated for the N-acetylaspartate+*N*-acetyl-aspartyl-glutamate (NAA + NAAG) resonance at 2.01 ppm, and the intensity of this resonance relative to the residual intensity was obtained (SNR). Mean (±SE) FWHM and SNR were 10.6 (±0.4) Hz and 12.1 (±0.4) Hz, respectively, for the baseline group (*n* = 17), and 14.6 (±0.8) Hz and 9.8 (±0.3) Hz, respectively, for the restraint group (*n* = 17). Individual metabolite concentrations were computed using the unsuppressed reference water signal for each individual scan. Cramér-Rao lower bound (CRLB) values were calculated by LCModel and reported as percent SD of each metabolite, as a measure of the reliability of the metabolite estimates.

The metabolites of interest were GABA (baseline CRLB: 12.7 ± 0.4%, restraint CRLB: 14.5 ± 0.6%) and Glu (baseline CRLB: 3.4 ± 0.1%, restraint CRLB: 3.9 ± 0.1%), the major neurotransmitters in the brain, as well as Gln (baseline CRLB: 9.7 ± 0.5%, restraint CRLB: 11.4 ± 0.3%), a neurotransmitter precursor, and combined Glu-Gln (Glx; baseline CRLB: 3.6 ± 0.1%, restraint CRLB: 4.2 ± 0.1%). To accurately extract the dominating metabolic changes observed before and after CRS, and to reduce systemic variations among studied animals, a relative quantification method, using an internal spectral reference was used. All ^1^H-MRS results presented here are expressed as a ratio to tCr (total creatine = Cr + PCr; baseline CRLB: 2.94 ± 0.06%, restraint CRLB: 3.12 ± 0.08%) spectral intensity, the simultaneously acquired internal reference peak (Block et al., 2009; Walter et al., 2009; Xu et al., 2013a).

#### Hippocampal volume

The three T2-weighted anatomic (coronal, sagittal and axial) data were preprocessed as above and then registered to the high-resolution atlas (no down-sampling). Atlas masks for bilateral hippocampus and whole-brain were used to automatically extract their respective volumes from the three T2-weighted anatomic images (coronal, sagittal, and axial). Hippocampal and whole-brain volumes from the three planes were averaged for each animal scan session. Spearman’s rank correlation method (*n* = 74) in RStudio 3.5.2 was used to determine the correlation of whole-brain volumes with the weight of animals at baseline because the volumes of the whole brain and several brain regions are known to increase with age in rats until they are two months old (Mengler et al., 2014). Hippocampal volume was normalized to the whole-brain volume (% whole-brain volume) to adjust for differences in head size (Welniak–Kaminska et al., 2019).

### Statistical analyses

For estimation based on confidence intervals (CIs), we directly introduced the raw data in https://www.estimationstats.com/ and downloaded the results and graphs. The paired differences for the comparisons are shown with Cumming estimation plots (Ho et al., 2019). The raw data are plotted on the upper axes. Each mean difference is plotted on the lower axes as a bootstrap sampling distribution and the 95% CIs are indicated by the ends of the vertical error bars; 5000 bootstrap samples were taken; the CI is bias-corrected and accelerated. To measure the effect size, we used unbiased Cohen’s *d* (also known as standardized mean difference). Paired median difference was used to measure the effect size of open arm entries and time spent within the open arms during the EPM test because many animals did not enter the open arms and the data are not normally distributed. The *p* values reported are the likelihoods of observing the effect sizes, if the null hypothesis of zero difference is true. For each permutation *p* value, 5000 reshuffles of the control and test labels were performed.

All comparisons were paired except for the rs-fMRI data (see explanation in Table 2). Statistically significant voxels from the rs-fMRI data analyses were used as a mask to extract the functional connectivity of each individual animal at each timepoint (current cohort only) from the GLM “parameter estimate” images from stage 2 of dual regression and from the contrast of parameter estimates image from first-level FEAT for seed-based analysis. These values were used to run unpaired estimation statistics as described above. Summary measurements (mean ± SD) are shown as gapped lines for each group. These functional connectivity measures were also used in the correlation analyses below.

Data from current cohort of animals which underwent imaging at both timepoints were used to correlate MRI measures to the behavioral measures. Spearman correlations (RStudio 3.5.2) between the following parameters were computed using data from both groups and timepoints: latency to first immobility behavior from FST data; connectivity (parameter estimates) of the salience network (ICA), interoceptive network (ICA), and cingulate cortex (seed-based) from the rs-fMRI data; Glx ratio from ^1^H-MRS data; and hippocampal volume (see Table 2). Pearson’s correlation method (*n* = 23) in RStudio 3.5.2 was used to determine correlation between baseline percentage hippocampal volume of the CRS group and postrestraint latency to first immobility behavior during FST.

## Results

### Increase in anxiety and depression-like behaviors

**Figure 3.**
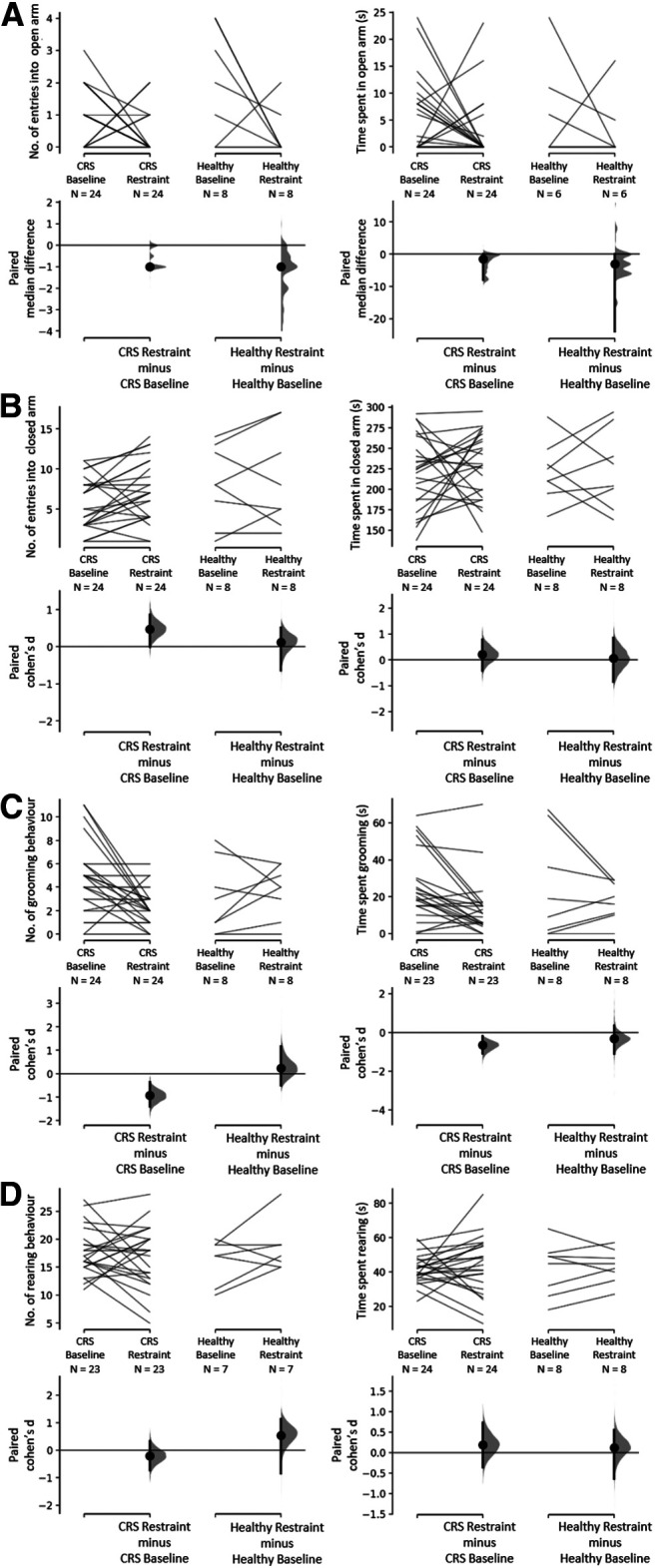
Effect of CRS on exploration in open (***A***) and closed (***B***) arms and on stress-response behaviors (***C–D***) displayed during EPM test. Exploration (open and closed arm entries and time spent) and stress-related behaviors (grooming and rearing) were measured for 5 min. ***A***, Total number of entries and time spent in open arms. The paired median differences for two comparisons are shown in the Cumming estimation plots. ***B***, Total number of entries and time spent in closed arms. ***C***, Number of grooming behaviors and time spent grooming. ***D***, Number of rearing behaviors and time spent rearing. The Cohen’s *d* for two comparisons are shown in the Cumming estimation plots. The raw data are plotted on the upper axes; each paired set of observations is connected by a line. On the lower axes, each paired difference is plotted as a bootstrap sampling distribution. Mean differences are depicted as dots; 95% CIs are indicated by the ends of the vertical error bars.

In the EPM test, there was a significant decrease in the number of entries into the open arms of the maze (baseline: 0.88 ± 0.18, *n* = 24; restraint: 0.32 ± 0.13, *n* = 24; median_diff_ = −1.0, *p *=* *0.0^aa^) and a significant increase in the number of entries in the closed arms of the maze (baseline: 5.63 ± 0.66, *n* = 24; restraint: 7.20 ± 0.74, *n* = 24; Cohen’s *d*  = 0.212, *p *=* *0.0282^ae^) following CRS. Note that there is considerable uncertainty about the magnitude of the effect of the restraint procedure on closed arms entries, with the CI stretching down toward negligible effects (95% CI[−0.0122, 0.856]; Fig. 3*B*). There were no significant differences for time spent exploring the open arms or the closed arms (Fig. 3*A*,*B* ^ac,ag^). There was also a significant decrease in the number of times the animals demonstrated grooming behaviors (baseline: 4.63 ± 0.64, *n* = 24; restraint: 2.28 ± 0.31, *n* = 24; Cohen’s *d* = −0.921, *p *=* *0.001^ai^; Fig. 3*C*) and the total time spent grooming (baseline: 24.70 ± 3.93 s, *n* = 23; restraint: 14.48 ± 3.30 s, *n* = 23; Cohen’s *d* = −0.642, *p *=* *0.0016^ak^; Fig. 3*C*). However, the number of times rats exhibited rearing behaviors remained similar between the two timepoints, as did total time spent rearing (Fig. 3*D*; Table 2 ^am,ao^). Healthy control animals which did not undergo the CRS procedure did not show any changes in any of the EPM measures between the two timepoints (Fig. 3; Table 2 ^aa-ap^).

There was no average change observed in the SPT data for both animals which underwent 13 d of restraint (baseline: 0.63 ± 0.03, *n* = 25; restraint: 0.69 ± 0.04, *n* = 25; 95% CI[−0.294, 0.889]; Cohen’s *d* = 0.289; *p *=* *0.312^aq^) and healthy control animals (baseline: 0.81 ± 0.03, *n* = 5; restraint: 0.78 ± 0.07, *n* = 5; 95% CI[−1.52, 0.831]; Cohen’s *d* = −0.191, *p *=* *0.807 ^ar^). Note that the CIs are large, and therefore, moderate effects in either direction cannot be ruled out.

**Figure 4.**
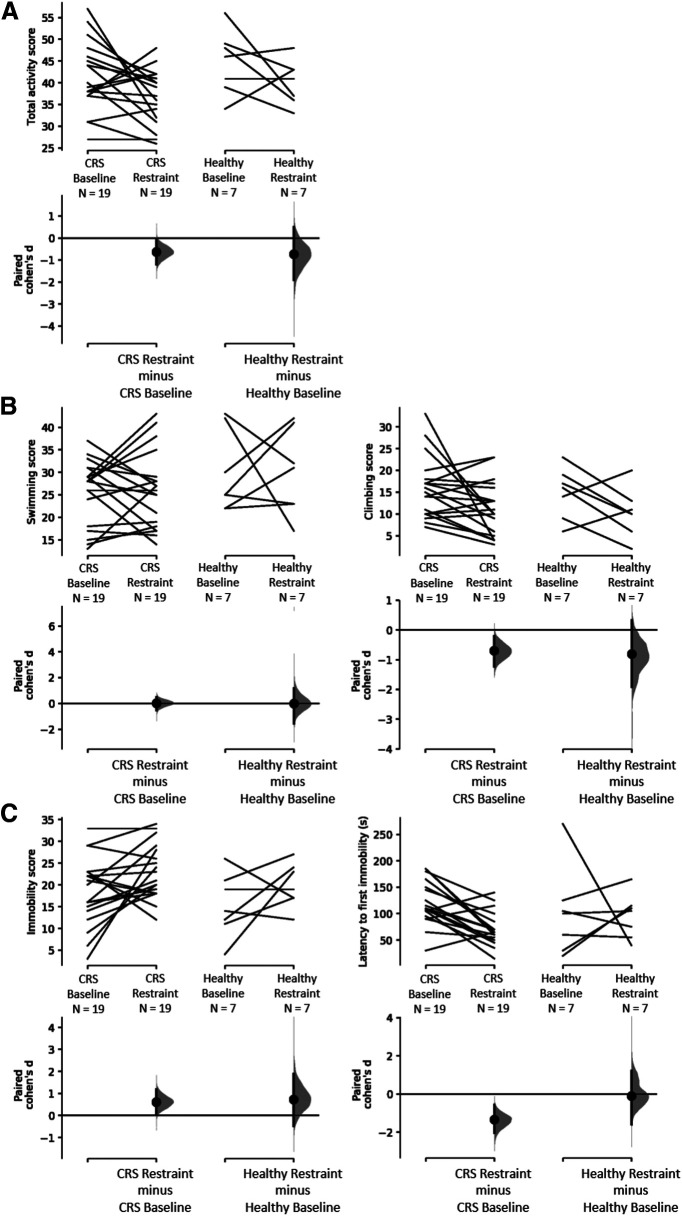
Effect of CRS on behaviors displayed during FST. Active behaviors (climbing and swimming) and immobility were measured for 5 min. ***A***, Decrease in active behaviors following 13 d of CRS and no change in the healthy control group. ***B***, No change in swimming scores in both groups and decrease in climbing in the CRS group only. ***C***, Increase in immobility and a decrease in time to first immobility behavior (known as latency time) following 13 d of CRS and no change in the healthy control group. The Cohen’s *d* for two comparisons are shown in the Cumming estimation plots. The raw data are plotted on the upper axes; each paired set of observations is connected by a line. On the lower axes, each paired mean difference is plotted as a bootstrap sampling distribution. Mean differences are depicted as dots; 95% CIs are indicated by the ends of the vertical error bars.

For the FST, animals in both groups had similar scores for immobility and climbing behaviors at baseline, displaying total active behaviors (climbing plus swimming) for ∼72% of the time (Fig. 4*A*; Table 2). After restraint, total activity decreased significantly compared with baseline (baseline: 42 ± 2, *n* = 19; restraint: 37 ± 1, *n* = 19; Cohen’s *d* = −0.62, *p *=* *0.0414^as^; Fig. 4*A*). When scores for both active behaviors were split, the decrease in climbing behaviors following restraint was statistically significant (baseline: 16 ± 2, *n* = 19; restraint: 11 ± 1, *n* = 19; Cohen’s *d* = −0.696, *p *=* *0.021^aw^), but not for swimming (baseline: 26 ± 2, *n* = 19; restraint: 26 ± 2, *n* = 19; Cohen’s *d* = 0.0269, *p *=* *0.896 ^au^; Fig. 4*B*). Additionally, immobility increased (baseline: 18 ± 2, *n* = 19; restraint: 23 ± 1, *n* = 19; Cohen’s *d* = 0.611, *p *=* *0.0438^ay^) and latency to first immobility behavior decreased (baseline: 119 ± 9 s, *n* = 19; restraint: 71 ± 7 s, *n* = 19; Cohen’s *d* = −1.34, *p *=* *0.0006^ba^; Fig. 4*C*). Healthy control animals which did not undergo the CRS procedure did not show changes in any of the FST measures between the two timepoints (Fig. 4; Table 2 ^as-bb^).

### Changes in resting-state functional connectivity

**Figure 5.**
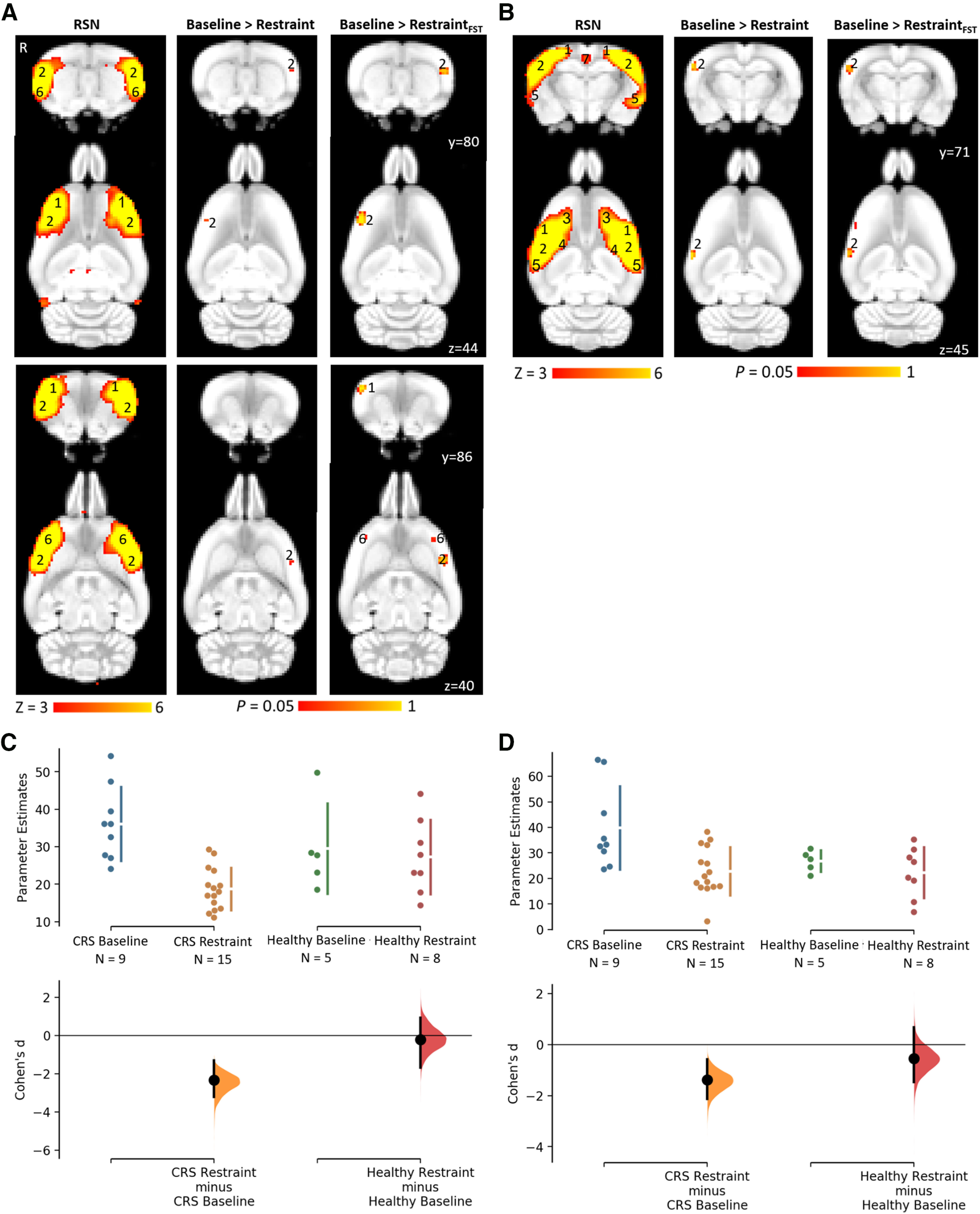
Decreased functional connectivity within the salience and interoceptive networks following CRS as detected by dual regression (***A***, ***B***) and corresponding Cumming estimation plots (***C***, ***D***). The figure illustrates coronal and corresponding axial slices of spatial statistical color-coded maps overlaid on the rat brain atlas (down-sampled by a factor of eight). ***A***, ***B***, Two RSNs (***A***, salience network; ***B***, interoceptive network) identified in the baseline rs-fMRI scans of six- to seven-week-old male Sprague Dawley rats under isoflurane-medetomidine anesthesia. The RSN maps are represented as *z* scores (*n* = 33, thresholded at *z* > 3), with a higher *z* score (yellow) representing a greater correlation between the time course of that voxel and the mean time course of the component. The changes in the functional connectivity within the two RSNs following 13 d of CRS are represented as *p* values (thresholded at *p *<* *0.05; baseline, *n* = 33; restraint, *n* = 15; restraint based on FST result, *n* = 9). R denotes right hemisphere. Significant clusters include various brain regions: 1, motor cortex; 2, somatosensory cortex; 3, frontal association cortex; 4, striatum/caudate putamen; 5, auditory cortex; 6, insular cortex; 7, retrosplenial cortex. The Cohen’s *d* for two comparisons are shown in the Cumming estimation plots below the associated statistical map (***C***, ***D***). The raw data are plotted on the upper axes; each mean difference is plotted on the lower axes as a bootstrap sampling distribution. Mean differences are depicted as dots; 95% CIs are indicated by the ends of the vertical error bars.

The interoceptive (Becerra et al., 2011; Seewoo et al., 2019) and salience (Bajic et al., 2016; Seewoo et al., 2019) networks were identified from baseline rs-fMRI data and used in dual regression analysis for detecting functional connectivity differences induced by CRS (Fig. 5). Dual regression analysis revealed a large decrease in connectivity of the bilateral somatosensory cortex to the salience network (baseline: 36 ± 3, *n* = 9; restraint: 19 ± 1, *n* = 15; unpaired Cohen’s *d* = −2.33, *p *=* *0.0^bc,be^; Fig. 5*A*,*C*) and of the right somatosensory cortex to the interoceptive network (baseline: 39 ± 5, *n* = 9; restraint: 23 ± 2, *n* = 15; unpaired Cohen’s *d* = −1.38, *p *=* *0.0032^bc,bg^; Fig. 5*B*,*D*). As a supplementary analysis, dual regression was conducted using a subset of the restraint group, which consisted of the nine animals exhibiting the greatest change in FST behavioral outcomes. A greater number of significant voxels with *p *<* *0.05^bd^ (both networks) and lower *p* values for changes in the salience network were obtained in the same brain regions (Fig. 5*A*,*B*; Table 3). Additionally, dual regression detected a significant decrease in connectivity of the right motor cortex and bilateral insular cortex to the salience network (Fig. 5*A*).

**Table 3:** Summary of changes in functional connectivity within the interoceptive and salience networks when using all rs-fMRI data from postrestraint timepoint versus using a subset of animals showing greatest behavioral changes in FST

RSN	Contrast	Minimum *p* value	Total number of significant voxels
Interoceptive network	Baseline > Restraint	0.005	32
	Baseline > Restraint_FST_	0.012	60
Salience network	Baseline > Restraint	0.029	11
	Baseline > Restraint_FST_	0.002	127

**Figure 6.**
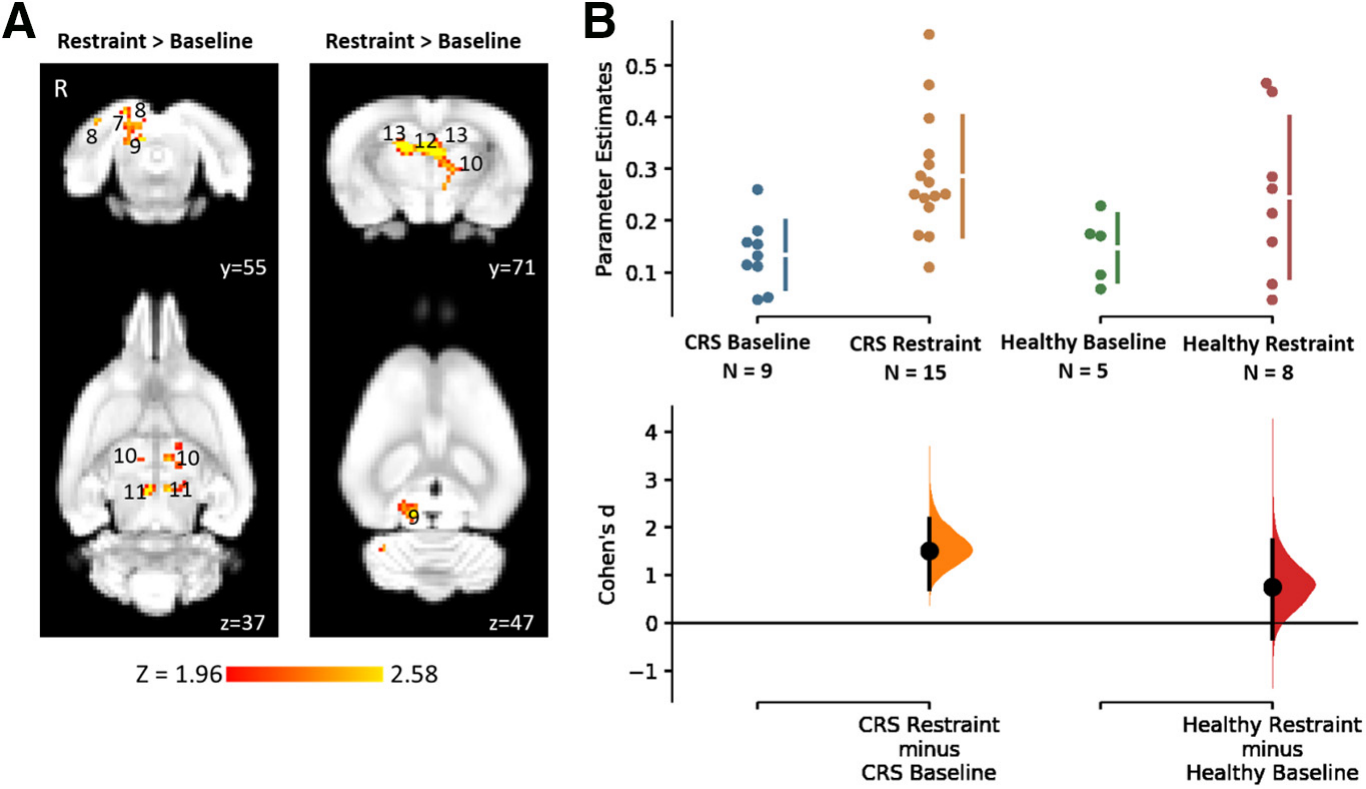
Increased functional connectivity to the cingulate cortex following CRS as detected by seed-based analysis (***A***) and corresponding Cumming estimation plots (***B***). The figure illustrates coronal and corresponding axial slices of spatial statistical color-coded maps overlaid on the rat brain atlas (down-sampled by a factor of eight). ***A***, Changes in the functional connectivity of the cingulate cortex between baseline and following 13 d of CRS as spatial color-coded Z (Gaussianized T/F) statistic images corrected for multiple comparisons at cluster level (thresholded at *p* < 0.05; baseline, *n* = 33; restraint, *n* = 15). R denotes right hemisphere. Significant clusters include various brain regions: 8, visual cortex; 9, inferior colliculus; 10, thalamus; 11, superior colliculus; 12, dentate gyrus; 13, CA3. The Cohen’s *d* for two comparisons are shown in the Cumming estimation plots below the associated statistical map (***B***). The raw data are plotted on the upper axes; each mean difference is plotted on the lower axes as a bootstrap sampling distribution. Mean differences are depicted as dots; 95% CIs are indicated by the ends of the vertical error bars.

When rs-fMRI data of all animals were analyzed using a seed-based analysis, a significantly greater functional connectivity of several brain regions to the cingulate cortex was detected in the restraint group (baseline: 0.13 ± 0.02, *n* = 9; restraint: 0.29 ± 0.03, *n* = 15; unpaired Cohen’s *d* = 1.51, *p *=* *0.0018^bj^; Fig. 6*B*). Specifically, hyperconnectivity was detected in the right retrosplenial cortex, visual cortex, and inferior colliculus and in the bilateral thalamus, superior colliculus, dentate gyrus, and cornu ammonis 3 (CA3; Fig. 6*A* ^bi^). Healthy control animals which did not undergo the CRS procedure did not show any change in functional connectivity between the two timepoints (Figs. 5*C*,*D*, 6*B*; Table 2 ^bf,bh,bk^).

### Changes in neurometabolite levels as detected by ^1^H-MRS

**Figure 7.**
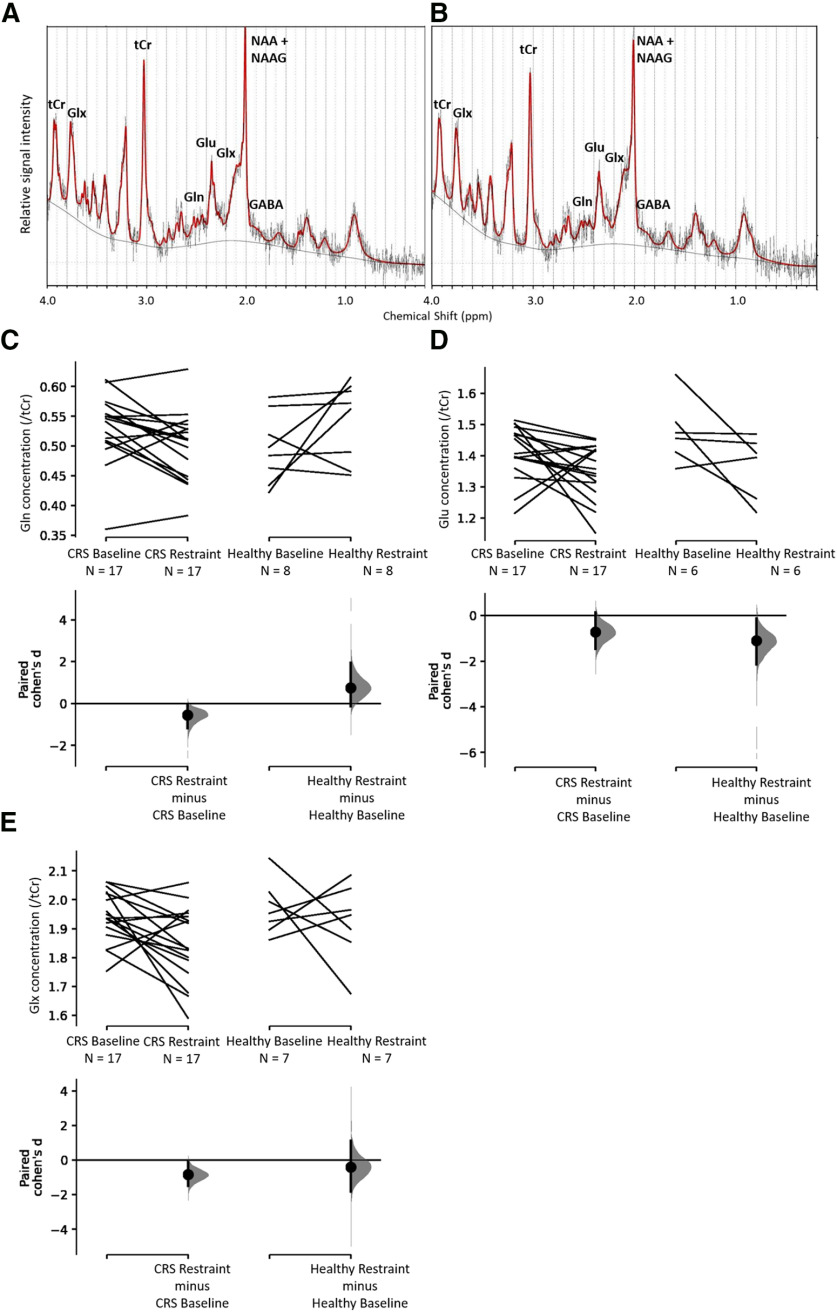
Representative spectra obtained from LCModel for proton magnetic resonance spectroscopy data at baseline (***A***) and restraint timepoints (***B***) for the CRS group and effect of restraint on Gln (***C***), Glu (***D***), and Glx (***E***). The figure shows spectra from a representative animal at baseline (***A***) and after 13 d of CRS (***B***) depicting longitudinally reproducible peaks of various metabolites quantified using the LCModel. ***C–E***, Cumming estimation plots showing paired Cohen’s *d* for two comparisons each. The raw data are plotted on the upper axes; each paired set of observations is connected by a line. On the lower axes, each paired mean difference is plotted as a bootstrap sampling distribution. Mean differences are depicted as dots; 95% CIs are indicated by the ends of the vertical error bars.

The concentrations of the neurotransmitters GABA and Glu, the neurotransmitter precursor Gln, and Glx were measured before and after CRS and were computed relative to tCr. Following restraint, rats had lower levels of Gln (baseline: 0.53 ± 0.01, *n* = 17; restraint: 0.50 ± 0.01, *n* = 17; Cohen’s *d* = −0.538, *p *=* *0.027^bl^), Glu (baseline: 1.41 ± 0.02, *n* = 17; restraint: 1.35 ± 0.02, *n* = 17; Cohen’s *d* = −0.711, *p *=* *0.071^bn^), and Glx (baseline: 1.94 ± 0.02, *n* = 17; restraint: 1.85 ± 0.03, *n* = 17; Cohen’s *d* = −0.84, *p *=* *0.0186 ^bp^) in the sensorimotor cortex (Fig. 7*C–E*). Note that there is considerable uncertainty about the magnitude of the effect of the restraint procedure on Glu levels, with the CI stretching up toward negligible effects (95% CI[−1.46, 0.134]; Fig. 7*D*). There was no change in GABA/tCr (baseline: 0.35 ± 0.01, *n* = 16; restraint: 0.34 ± 0.01, *n* = 16^br^) and Gln/Glu (baseline: 0.38 ± 0.01, *n* = 17; restraint: 0.37 ± 0.01, *n* = 17^bt^). Healthy control animals which did not undergo the CRS procedure did not show any change in neurometabolite levels between the two timepoints (Fig. 7*C-E*; Table 2 ^bl-bu^).

### Change in hippocampal volume

**Figure 8.**
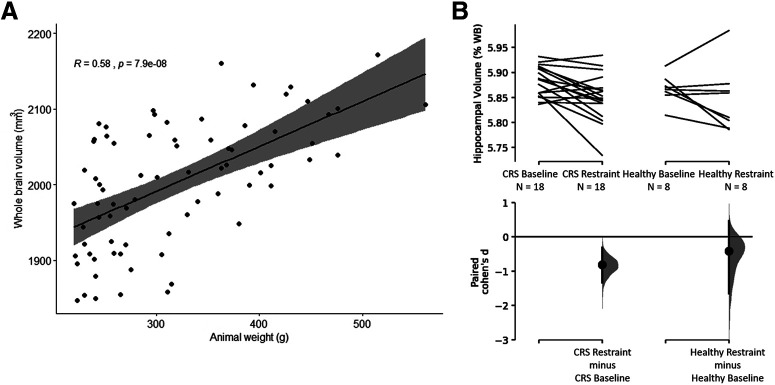
Correlation between weight of animals and whole-brain volume at baseline (***A***) and percentage hippocampal volume before and after CRS (***B***). ***A***, Whole-brain volumes (mm^3^) plotted against the animal’s weight at baseline (*n* = 74). Correlation was determined using Spearman’s rank correlation method. In ***B***, hippocampal volumes were calculated as a percentage of whole-brain volume. ***B***, Decrease in percentage hippocampal volume following 13 d of CRS and no change in the healthy control group. The Cohen’s *d* for two comparisons are shown in the Cumming estimation plots. The raw data are plotted on the upper axes; each paired set of observations is connected by a line. On the lower axes, each paired mean difference is plotted as a bootstrap sampling distribution. Mean differences are depicted as dots; 95% CIs are indicated by the ends of the vertical error bars.

Spearman’s rank correlation method revealed a significant correlation of whole-brain volumes with the weight of the animals at baseline (*S* = 28 632, *R* = 0.58, *p* = 7.909e^−8bv^; Fig. 8*A*), with the mean whole-brain volume to body weight ratio of the Sprague Dawley rats being 6.64 ± 0.16 mm^3^/g. Percentage hippocampal volume decreased following CRS (baseline: 5.88 ± 0.01, *n* = 18; restraint: 5.85 ± 0.01, *n* = 18; Cohen’s *d* = −0.811; *p *=* *0.003^bw^; Fig. 8*B*) but did not change significantly in healthy controls (baseline: 5.87 ± 0.01, *n* = 8; restraint: 5.85 ± 0.02, *n* = 8^bx^; Fig. 8*B*).

### Correlations

**Figure 9.**
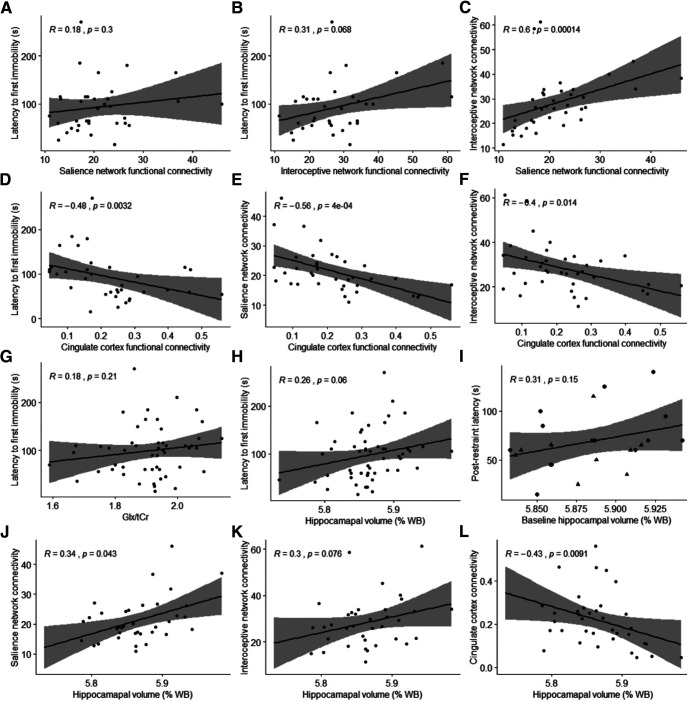
Correlations between behavioral tests and MRI measures. In ***A–G***, Comparisons of the following parameters from both CRS and healthy control groups at both timepoints were made by Spearman correlations: latency time from FST data; connectivity (average parameter estimates) of the salience network, interoceptive network and cingulate cortex from the rs-fMRI data; and Glx/tCr ratio from ^1^H-MRS data (no multiple comparison correction). In ***H–L***, hippocampal volumes were calculated as a percentage of whole-brain volume and compared to latency time from FST data (***H***, ***I***), and functional brain connectivity (***J–L***) (no multiple comparison correction). In ***I***, Pearson’s correlation was performed between baseline percentage hippocampal volume of CRS group and postrestraint latency to first immobility behavior during FST of the same animals (baseline, *n* = 23; restraint, *n* = 23). Data points with triangular shape represent the nine animals, which were used for FST-based ICA/dual regression analysis.

Spearman’s rank correlation test using data from both groups and timepoints revealed significant correlations between latency to first immobility behavior and several MRI measures as well as between different MRI measures (Table 2 ^by-cj^; Fig. 9). However, only the correlation and of the salience network connectivity with the interoceptive network and cingulate cortex connectivity and of the cingulate cortex connectivity with latency to first immobility behavior survived multiple comparison correction. A Pearson’s correlation test revealed no significant correlation between baseline percentage hippocampal volume of the CRS group and postrestraint latency to first immobility behavior during FST (*R* = 0.311, *t*_(21)_ = 1.50, *p *=* *0.148^cg^; Fig. 9*I*).

## Discussion

Animal models are an indispensable tool for studying etiology, progress, and treatment of depression in a controlled environment. However, there remains controversy regarding the validity of using rodent models of human neuropsychiatric disorders. Prior work in rodents investigating anxiety and depression-like behaviors (Suvrathan et al., 2010; Ulloa et al., 2010; Chiba et al., 2012; Bogdanova et al., 2013), peripheral biomarkers, functional connectivity of the brain (Henckens et al., 2015), and hippocampal volume (Lee et al., 2009; Alemu et al., 2019) supports the validity of the CRS paradigm as a depression model (Wang et al., 2017). However, our study is the first to correlate MRI measures of functional, chemical, and structural changes in the brain with abnormal behavior in the CRS model. We note that most of the correlation measures did not survive multiple comparison corrections and should therefore be interpreted with caution. Nevertheless, similarities between our data and the MRI outcomes in humans suggest that the CRS model may be a useful component of translational studies aimed at developing and refining novel treatments for depression in humans.

### Aberrant resting-state functional connectivity following CRS

One of the most consistent pathophysiologies of depression that has emerged from rs-fMRI studies is the abnormal regulation of the cortico-limbic mood regulating circuits. The human salience and interoceptive networks play an important role in being aware of, and orienting and responding to, biologically relevant stimuli (Harshaw, 2015), while the DMN is implicated in rumination, self-referential functions, and episodic memory retrieval (Lu et al., 2012). Because these distributed neuronal networks encompassing cortical and limbic brain regions normally regulate aspects of emotional behavior, the dysregulation of functional connectivity within these networks is known to be associated with depression (Wang et al., 2012; Helm et al., 2018).

ICA and dual regression analysis of our rodent rs-fMRI data detected decreased functional connectivity of the bilateral somatosensory cortex to the salience network, and of the right somatosensory cortex to the interoceptive network following CRS, and the reductions in connectivity within the two RSNs were strongly correlated to each other. This is in accordance with previous studies reporting altered functional connectivity in both the salience and interoceptive networks in humans with depression compared with healthy individuals (Manoliu et al., 2014; Harshaw, 2015). For example, Yin et al. (2018) observed decreased functional connectivity of insular cortex to somatosensory and motor cortices in patients with bipolar disorder in the period of depression.

The decrease in functional connectivity within the salience and interoceptive networks is known to be associated with negative response biases in patients with depression and correlated to their severity of symptoms (Manoliu et al., 2014; Harshaw, 2015). In animals, immobility and latency to first immobility behavior in FST are believed to reflect a failure to persist in escape-directed behavior after stress and have been suggested to represent “behavioral despair” (Slattery and Cryan, 2012). These measures are consistently used as a preclinical screen for antidepressants and antidepressants that are effective in humans are found to decrease immobility in rats in FST (Cryan et al., 2005). However, salience and interoceptive network connectivity were not correlated with latency to first immobility behavior during FST in our rats. When a subset of animals showing the greatest depression-like behaviors in the FST was used in dual regression analysis, a decrease in connectivity of the right motor cortex and bilateral insular and somatosensory cortices to the salience network was also detected. Therefore, despite being a smaller group, the use of a subset of animals selected based on their FST performance resulted in increased sensitivity of the dual regression tool in detecting between-timepoint differences, showing some correlation between functional connectivity and behavior. CRS may induce abnormal behavioral responses in animals as a result of insular dysfunction within the salience network leading to an abnormal switching between the DMN and the central executive network (Manoliu et al., 2014).

The DMN plays an important role in the pathophysiology of depression (Sheline et al., 2009; Zhu et al., 2012). One critical element of the DMN is the cingulate cortex, which has increased connectivity with other limbic areas in patients with depression (Greicius et al., 2007; Sheline et al., 2009; Davey et al., 2012; Fang et al., 2012; Rolls et al., 2019). The results of the current study are consistent with these data; we detected hyperconnectivity of the cingulate cortex to the right retrosplenial cortex, visual cortex, inferior colliculus, bilateral thalamus, superior colliculus, and hippocampus following CRS. Additionally, cingulate cortex connectivity was very strongly correlated with behavioral despair (latency in FST) in our rats. Cingulate cortex connectivity plays a significant role in clinical symptoms (Walter et al., 2009), with higher functional connectivity leading to dysfunctional emotion, internal inspection, and endocrine regulation (Fang et al., 2012). For example, increased functional connectivity between the thalamus and the cingulate cortex may result from increased emotional processing, at the cost of executive functions (Greicius et al., 2007). However, our behavioral tests did not specifically address executive functioning in rats, and this could be addressed in future studies using appropriate cognitive tests.

Our rs-fMRI findings differ from a previous animal study using a shorter CRS protocol (2 h/d for 10 d), which did not find any significant changes in the RSNs despite using the same ICA/dual regression approach of rs-fMRI data analysis performed here (Henckens et al., 2015). Moreover, when comparing “overall connectivity strength,” connectivity was increased in somatosensory and visual networks, which was not observed in our experiments. The shorter duration of the restraint stress, as well as intrinsic differences between the rs-fMRI data analysis methods used to detect changes in connectivity, and the effect of an isoflurane-only anesthetic protocol on RSNs (Paasonen et al., 2018) used in the previous study (Henckens et al., 2015) could be the cause of these inconsistencies.

Comparison of the hyperconnectivity observed here to findings in other animal models used to investigate depression and anxiety is interesting. Brain activation in cortical and hippocampal regions in mice following chronic social defeat stress is observed in manganese-enhanced MRI (Laine et al., 2017). Additionally, aberrant hippocampal, thalamic, and cortical connectivity is reported in other rodent models using different data acquisition and/or analysis methods. For example, in the chronic unpredictable stress rat model, rs-fMRI studies found increased functional connectivity of the hippocampus to several brain regions (Magalhães et al., 2019), increased functional connectivity between atrophied brain regions such as the hippocampus, striatum and cingulate, motor and somatosensory cortices (Magalhães et al., 2018), and increased regional homogeneity (coherence of intraregional spontaneous low-frequency activity) in the hippocampus, thalamus and visual cortex as well as a decreased regional homogeneity in the motor cortex (Li et al., 2018). Electrophysiology studies have also reported long-lasting inhibition of long-term potentiation in the thalamo–cortical circuitry (Zheng et al., 2012) and in the hippocampal–cortical circuitry (Cerqueira et al., 2007) in chronic unpredictable stress models while hippocampal–cortical circuitry inhibition was also reported in acute platform stress rat models (Rocher et al., 2004). These different animal models reflect specific aspects of depression and therefore, they may be useful for understanding the heterogeneity of human depression.

### Decrease in Glu and Gln levels following CRS

Several preclinical and clinical studies have proposed that altered glutamatergic neurotransmission plays a pivotal role in the pathogenesis of mood disorders (Sanacora et al., 2012; Marrocco et al., 2014; Moriguchi et al., 2019). Accordingly, another major finding of the present study was the significant decreases in Gln, Glu, and Glx in the left sensorimotor cortex following CRS. Human ^1^H-MRS studies have reproducibly reported a reduced concentration in Glu, Gln, and/or Glx in several brain regions including the anterior cingulate cortex (Mirza et al., 2004; Luykx et al., 2012) and the prefrontal cortex (Hasler et al., 2007; Portella et al., 2011). Similarly, other ^1^H-MRS studies of animal models of depression such as the chronic mild stress and the chronic social isolation models have reported decreases in these neurometabolites in the prefrontal cortex (Hemanth Kumar et al., 2012) and hippocampus (Hemanth Kumar et al., 2012; Shao et al., 2015).

The majority of the measured neurometabolites are intracellular, with a small portion reflecting synaptic Glu, therefore to infer changes in glutamatergic neurotransmission from ^1^H-MRS studies is difficult (Sanacora et al., 2012). Nevertheless, a change in Glu-related neurometabolite concentration may reflect a change in Glu–Gln cycling or overall Glu metabolism (Yildiz-Yesiloglu and Ankerst, 2006). The foremost metabolic pathway of Glu is the synthesis of Gln in glial cells from Glu, the transport of Gln to nerve cell terminals, the conversion of Gln into the neurotransmitter Glu, the release of Glu and the final re-uptake of Glu by the glia (Pfleiderer et al., 2003). Since the measured neurometabolites largely represent the intracellular pool contained in glutamatergic neurons and glia, a decrease in Glu, Gln, and Glx may reflect an impairment of the neuron–astrocyte integrity, energy metabolism, glial cell dysfunction, or a loss of glial cells, particularly astrocytes (Yildiz-Yesiloglu and Ankerst, 2006; Lee et al., 2013).

The shortage in these neurometabolites might be because of a reduction in the number of astrocytes which in turn alters neuronal activity and therefore may contribute to depression-like behaviors, as previously shown in an L-α aminoadipic acid (L-AAA) infusion mouse model (Lee et al., 2013). However, there was also no correlation between Glx levels and depression-like behaviors post-CRS in the present study. This is surprising because a relatively recent meta-analysis on Glx concentrations in depression found that decrease in Glx in patients with depression was positively associated with depression severity (Arnone et al., 2015). While the functional connectivity within the DMN increased post-CRS in our animals, there were also no changes in Gln/Glu ratio in either the CRS or healthy control groups, suggesting the absence of change in glutamatergic activity and therefore, the absence of Glu-related excitotoxicity in the cortex of our animals.

### Decrease in hippocampal volume

There are several convergent lines of evidence from both preclinical and clinical studies that implicate the hippocampus in the pathogenesis of depression (Campbell and Macqueen, 2004). The hippocampus is a key brain region within the limbic system and plays a determinant role in emotional regulation. As mentioned above, the hippocampus is one of several regions, including the prefrontal cortex, the cingulate cortex, and the thalamus that have been identified to be part of the DMN showing abnormally higher functional connectivity in patients with depression compared with healthy individuals (Sheline et al., 2009). Additionally, the hippocampus is known to be a highly stress-sensitive structure as increased levels of glucocorticoids in stressful situations are known to disrupt hippocampal neurogenesis, which may lead to hippocampal atrophy (Dranovsky and Hen, 2006). A reduction in hippocampal volume has been consistently associated with depression in humans (McKinnon et al., 2009). However, the stage at which hippocampal atrophy begins in human depression is unclear and so is the direction of causality.

There are two main hypotheses regarding how depression is associated with hippocampal atrophy. First, hippocampal volume reduction, probably as a result of early life adversity, poverty, and stress, might predispose people to depression. This hypothesis seems consistent with smaller hippocampal volumes already present in first depressive episodes (Cole et al., 2011) and in young children (Barch et al., 2019) and adolescents (Rao et al., 2010) with depression. The second hypothesis, known as the neurotoxicity hypothesis, suggests that cumulative exposure to disrupted emotion regulation, stress reactivity, glucocorticoids, and antidepressant medications as a result of depression increases neuronal susceptibility to insults and therefore leads to hippocampal deficits (Sheline, 2011). This hypothesis is consistent with hippocampal atrophy being more pronounced among individuals with recurrent episodes and in chronic depression (McKinnon et al., 2009; Cheng et al., 2010; Brown et al., 2014).

The longitudinal nature of the present study precludes the first hypothesis in CRS animals. While hippocampal volume was weakly correlated with latency overall, there was no correlation between baseline hippocampal volume and post-CRS latency. This shows that baseline hippocampal volume did not predict severity of symptoms in this CRS model. Therefore, this study supports the neurotoxicity hypothesis and further suggests that the reduction in hippocampal volume might happen at a very early stage in depression, i.e., within only three weeks in this animal model. Additionally, hippocampal volume was correlated to functional connectivity of the salience network, interoceptive network, and cingulate cortex, which suggests the presence of a common pathway for the mechanism of depression.

### Study limitations

Our study has four main limitations. First, only young adult male rats were used in this study, although CRS has been shown to successfully induce depression-like behaviors in freely cycling adolescent female rats (Hibicke et al., 2017a,b). Future studies could expand the applicability of present results by investigating brain changes following CRS in female rats and in older rats. Second, the SPT did not detect anhedonia in our animals following restraint, despite anhedonia being a well-documented effect of CRS (Chiba et al., 2012; Ampuero et al., 2015; Liu et al., 2016). Use of non-acidified water and longer habituation and/or test times as performed in these studies may be required. Third, MRI data were acquired under anesthesia, which could potentially alter the blood oxygen level-dependent (BOLD) signal detection. However, functional connectivity patterns of animals anaesthetized using a combination of low-dose isoflurane and medetomidine have good correspondence with those of awake rats (Paasonen et al., 2018) with strong intercortical and cortical-subcortical functional connectivity (Grandjean et al., 2014; Bukhari et al., 2017) and are reproducible (Lu et al., 2012). Moreover, ^1^H-MRS data were acquired only in the left sensorimotor cortex. Future studies can investigate neurometabolite changes in bilateral sensorimotor cortex as well as in other brain regions such as the basal ganglia, hippocampus, anterior cingulate cortex, and occipital cortex, which are extensively investigated in ^1^H-MRS studies of human depression. Neurometabolite and structural changes could be confirmed using invasive methods following CRS. Finally, the pharmacological or interventional validity of the present neuroimaging findings is unknown. Future work should examine the utility of these findings as preclinical target engagement biomarkers with pharmacological and neuromodulatory interventions. If this proves to be the case, this animal model has potential utility for high throughput dose finding studies of neurotherapeutics and novel interventions.

## Conclusion

The present study is the first to demonstrate significant changes in functional connectivity, neurometabolite levels, and hippocampal volume in the same young adult male rats post-CRS and the correlation of these measures with changes in behavior provide insight into the neurobiological changes that may underpin patient symptoms. Cumulative exposure to stress might increases neuronal and astrocytic death leading to hippocampal atrophy and a shortage in Glu and Gln, which in turn alters neuronal activity and therefore contribute to learned helplessness. Overall, the substantial concordance of the present findings with the literature of human depression presents a unique opportunity for the integration of behavioral, cellular and molecular changes detected in this depression model with changes in MRI measures of brain function, chemistry and structure that may be translated to future studies of the human disorder, especially when testing the effects of new drug treatments or therapies.

## Synthesis

Reviewing Editor: Aniko Korosi, University of Amsterdam Decisions are customarily a result of the Reviewing Editor and the peer reviewers coming together and discussing their recommendations until a consensus is reached. When revisions are invited, a fact-based synthesis statement explaining their decision and outlining what is needed to prepare a revision will be listed below. The following reviewer(s) agreed to reveal their identity: Juan Nácher.

Reviewer 1

The authors have answered satisfactorily all my queries.

This is a very interesting and novel study. There are very few longitudinal studies on the effects of chronic stress. These types of studies are very relevant because they will allow the comparison with human data, especially with that obtained from psychiatric disorders in which stress can be a precipitating factor.

## References

[B1] Alemu JL, Elberling F, Azam B, Pakkenberg B, Olesen MV (2019) Electroconvulsive treatment prevents chronic restraint stress-induced atrophy of the hippocampal formation-a stereological study. Brain Behav 9:e01195. 10.1002/brb3.1195 30656862 PMC6379514

[B2] Ampuero E, Luarte A, Santibañez M, Varas-Godoy M, Toledo J, Diaz-Veliz G, Cavada G, Rubio FJ, Wyneken U (2015) Two chronic stress models based on movement restriction in rats respond selectively to antidepressant drugs: aldolase C as a potential biomarker. Int J Neuropsychopharmacol 18:pyv038. 10.1093/ijnp/pyv038 25813018 PMC4648154

[B3] Arnone D, Mumuni AN, Jauhar S, Condon B, Cavanagh J (2015) Indirect evidence of selective glial involvement in glutamate-based mechanisms of mood regulation in depression: meta-analysis of absolute prefrontal neuro-metabolic concentrations. Eur Neuropsychopharmacol 25:1109–1117. 10.1016/j.euroneuro.2015.04.016 26028038

[B4] Bajic D, Craig MM, Borsook D, Becerra L (2016) Probing intrinsic resting-state networks in the infant rat brain. Front Behav Neurosci 10:192. 10.3389/fnbeh.2016.00192 27803653 PMC5067436

[B5] Barch DM, Tillman R, Kelly D, Whalen D, Gilbert K, Luby JL (2019) Hippocampal volume and depression among young children. Psychiatry Res Neuroimaging 288:21–28. 10.1016/j.pscychresns.2019.04.012 31071541 PMC6550342

[B6] Becerra L, Pendse G, Chang P-C, Bishop J, Borsook D (2011) Robust reproducible resting state networks in the awake rodent brain. PLoS One 6:e25701. 10.1371/journal.pone.0025701 22028788 PMC3196498

[B7] Beckmann CF, Jenkinson M, Smith SM (2003) General multilevel linear modeling for group analysis in fMRI. Neuroimage 20:1052–1063. 10.1016/S1053-8119(03)00435-X 14568475

[B8] Beckmann CF, DeLuca M, Devlin JT, Smith SM (2005) Investigations into resting-state connectivity using independent component analysis. Philos Trans R Soc Lond B Biol Sci 360:1001–1013. 10.1098/rstb.2005.1634 16087444 PMC1854918

[B9] Bidgood WD Jr, Horii SC, Prior FW, Van Syckle DE (1997) Understanding and using DICOM, the data interchange standard for biomedical imaging. J Am Med Inform Assoc 4:199–212. 10.1136/jamia.1997.0040199 9147339 PMC61235

[B10] Block W, Träber F, von Widdern O, Metten M, Schild H, Maier W, Zobel A, Jessen F (2009) Proton MR spectroscopy of the hippocampus at 3 T in patients with unipolar major depressive disorder: correlates and predictors of treatment response. Int J Neuropsychopharmacol 12:415–422. 10.1017/S1461145708009516 18845018

[B11] Bogdanova OV, Kanekar S, D'Anci KE, Renshaw PF (2013) Factors influencing behavior in the forced swim test. Physiol Behav 118:227–239. 10.1016/j.physbeh.2013.05.012 23685235 PMC5609482

[B12] Bravo JA, Díaz-Veliz G, Mora S, Ulloa JL, Berthoud VM, Morales P, Arancibia S, Fiedler JL (2009) Desipramine prevents stress-induced changes in depressive-like behavior and hippocampal markers of neuroprotection. Behav Pharmacol 20:273–285. 10.1097/FBP.0b013e32832c70d9 19424057

[B13] Brown ES, Hughes CW, McColl R, Peshock R, King KS, Rush AJ (2014) Association of depressive symptoms with hippocampal volume in 1936 adults. Neuropsychopharmacology 39:770–779. 10.1038/npp.2013.271 24220026 PMC3895255

[B14] Bukhari Q, Schroeter A, Cole DM, Rudin M (2017) Resting state fMRI in mice reveals anesthesia specific signatures of brain functional networks and their interactions. Front Neural Circuits 11:5. 10.3389/fncir.2017.00005 28217085 PMC5289996

[B15] Buyukdura JS, McClintock SM, Croarkin PE (2011) Psychomotor retardation in depression: biological underpinnings, measurement, and treatment. Prog Neuropsychopharmacol Biol Psychiatry 35:395–409. 10.1016/j.pnpbp.2010.10.019 21044654 PMC3646325

[B16] Campbell S, Macqueen G (2004) The role of the hippocampus in the pathophysiology of major depression. J Psychiatry Neurosci 29:417–426. 15644983 PMC524959

[B17] Cerqueira JJ, Mailliet F, Almeida OFX, Jay TM, Sousa N (2007) The prefrontal cortex as a key target of the maladaptive response to stress. J Neurosci 27:2781–2787. 10.1523/JNEUROSCI.4372-06.2007 17360899 PMC6672565

[B18] Cheng YQ, Xu J, Chai P, Li HJ, Luo CR, Yang T, Li L, Shan BC, Xu XF, Xu L (2010) Brain volume alteration and the correlations with the clinical characteristics in drug-naïve first-episode MDD patients: a voxel-based morphometry study. Neurosci Lett 480:30–34. 10.1016/j.neulet.2010.05.075 20594947

[B19] Chiba S, Numakawa T, Ninomiya M, Richards MC, Wakabayashi C, Kunugi H (2012) Chronic restraint stress causes anxiety- and depression-like behaviors, downregulates glucocorticoid receptor expression, and attenuates glutamate release induced by brain-derived neurotrophic factor in the prefrontal cortex. Prog Neuropsychopharmacol Biol Psychiatry 39:112–119. 10.1016/j.pnpbp.2012.05.018 22664354

[B20] Cole J, Costafreda SG, McGuffin P, Fu CHY (2011) Hippocampal atrophy in first episode depression: a meta-analysis of magnetic resonance imaging studies. J Affect Disord 134:483–487. 10.1016/j.jad.2011.05.057 21745692

[B21] Cryan JF, Valentino RJ, Lucki I (2005) Assessing substrates underlying the behavioral effects of antidepressants using the modified rat forced swimming test. Neurosci Biobehav Rev 29:547–569. 10.1016/j.neubiorev.2005.03.008 15893822

[B22] Davey CG, Harrison BJ, Yücel M, Allen NB (2012) Regionally specific alterations in functional connectivity of the anterior cingulate cortex in major depressive disorder. Psychol Med 42:2071–2081. 10.1017/S0033291712000323 22954259

[B23] Dranovsky A, Hen R (2006) Hippocampal neurogenesis: regulation by stress and antidepressants. Biol Psychiatry 59:1136–1143. 10.1016/j.biopsych.2006.03.082 16797263 PMC7537828

[B24] Drevets WC, Savitz J, Trimble M (2008) The subgenual anterior cingulate cortex in mood disorders. CNS Spectr 13:663–681. 10.1017/s1092852900013754 18704022 PMC2729429

[B25] Fang P, Zeng L-L, Shen H, Wang L, Li B, Liu L, Hu D (2012) Increased cortical-limbic anatomical network connectivity in major depression revealed by diffusion tensor imaging. PLoS One 7:e45972 10.1371/journal.pone.0045972 23049910 PMC3458828

[B26] Grandjean J, Schroeter A, Batata I, Rudin M (2014) Optimization of anesthesia protocol for resting-state fMRI in mice based on differential effects of anesthetics on functional connectivity patterns. Neuroimage 102:838–847. 10.1016/j.neuroimage.2014.08.043 25175535

[B27] Greicius MD, Flores BH, Menon V, Glover GH, Solvason HB, Kenna H, Reiss AL, Schatzberg AF (2007) Resting-state functional connectivity in major depression: abnormally increased contributions from subgenual cingulate cortex and thalamus. Biol Psychiatry 62:429–437. 10.1016/j.biopsych.2006.09.020 17210143 PMC2001244

[B28] Harshaw C (2015) Interoceptive dysfunction: toward an integrated framework for understanding somatic and affective disturbance in depression. Psychol Bull 141:311–363. 10.1037/a0038101 25365763 PMC4346391

[B29] Hasler G, van der Veen JW, Tumonis T, Meyers N, Shen J, Drevets WC (2007) Reduced prefrontal glutamate/glutamine and γ-aminobutyric acid levels in major depression determined using proton magnetic resonance spectroscopy. Arch Gen Psychiatry 64:193–200. 10.1001/archpsyc.64.2.193 17283286

[B30] Helm K, Viol K, Weiger TM, Tass PA, Grefkes C, Del Monte D, Schiepek G (2018) Neuronal connectivity in major depressive disorder: a systematic review. Neuropsychiatr Dis Treat 14:2715–2737. 10.2147/NDT.S170989 30425491 PMC6200438

[B31] Hemanth Kumar BS, Mishra SK, Rana P, Singh S, Khushu S (2012) Neurodegenerative evidences during early onset of depression in CMS rats as detected by proton magnetic resonance spectroscopy at 7 T. Behav Brain Res 232:53–59. 10.1016/j.bbr.2012.03.011 22449862

[B32] Henckens M, van der Marel K, van der Toorn A, Pillai AG, Fernández G, Dijkhuizen RM, Joëls M (2015) Stress-induced alterations in large-scale functional networks of the rodent brain. Neuroimage 105:312–322. 10.1016/j.neuroimage.2014.10.037 25462693

[B33] Hibicke M, Graham MA, Hayslett RL (2017a) Adolescent chronic restraint stress (aCRS) elicits robust depressive-like behavior in freely cycling, adult female rats without increasing anxiety-like behaviors. Exp Clin Psychopharmacol 25:74–83. 10.1037/pha0000119 28287791

[B34] Hibicke M, Graham MA, Hayslett RL (2017b) Adult female rats exposed to adolescent chronic restraint stress (aCRS) display robust depressive-like behavior, cognitive impairment, and an unexpected correlation with serum BDNF. FASEB J 31:989–984.27895108

[B35] Ho J, Tumkaya T, Aryal S, Choi H, Claridge-Chang A (2019) Moving beyond P values: data analysis with estimation graphics. Nat Methods 16:565–566. 10.1038/s41592-019-0470-3 31217592

[B36] Ilamkar KR (2014) Psychomotor retardation, attention deficit and executive dysfunctional in young non-hospitalised un-medicated non-psychotic unipolar depression patients. J Clin Diagn Res 8:124–126. 10.7860/JCDR/2014/7221.4026 24701501 PMC3972527

[B37] Jenkinson M, Smith S (2001) A global optimisation method for robust affine registration of brain images. Med Image Anal 5:143–156. 10.1016/s1361-8415(01)00036-6 11516708

[B38] Jenkinson M, Bannister P, Brady M, Smith S (2002) Improved optimization for the robust and accurate linear registration and motion correction of brain images. Neuroimage 17:825–841. 10.1016/s1053-8119(02)91132-8 12377157

[B39] Jenkinson M, Beckmann CF, Smith SM, Woolrich MW, Behrens TEJ (2012) FSL. Neuroimage 62:782–790. 10.1016/j.neuroimage.2011.09.015 21979382

[B40] Kjonigsen LJ, Lillehaug S, Bjaalie JG, Witter MP, Leergaard TB (2015) Waxholm Space atlas of the rat brain hippocampal region: three-dimensional delineations based on magnetic resonance and diffusion tensor imaging. Neuroimage 108:441–449. 10.1016/j.neuroimage.2014.12.080 25585022

[B41] Laine MA, Sokolowska E, Dudek M, Callan S-A, Hyytiä P, Hovatta I (2017) Brain activation induced by chronic psychosocial stress in mice. Sci Rep 7:15061. 10.1038/s41598-017-15422-5 29118417 PMC5678090

[B42] Lee T, Jarome T, Li S-J, Kim JJ, Helmstetter FJ (2009) Chronic stress selectively reduces hippocampal volume in rats: a longitudinal magnetic resonance imaging study. Neuroreport 20:1554–1558. 10.1097/WNR.0b013e328332bb09 19858767 PMC2783199

[B43] Lee Y, Son H, Kim G, Kim S, Lee DH, Roh GS, Kang SS, Cho GJ, Choi WS, Kim HJ (2013) Glutamine deficiency in the prefrontal cortex increases depressive-like behaviours in male mice. J Psychiatry Neurosci 38:183–191. 10.1503/jpn.120024 23031251 PMC3633711

[B44] Lener MS, Niciu MJ, Ballard ED, Park M, Park LT, Nugent AC, Zarate CA Jr (2017) Glutamate and gamma-aminobutyric acid systems in the pathophysiology of major depression and antidepressant response to ketamine. Biol Psychiatry 81:886–897. 10.1016/j.biopsych.2016.05.005 27449797 PMC5107161

[B45] Li J, Yang R, Xia K, Wang T, Nie B, Gao K, Chen J, Zhao H, Li Y, Wang W (2018) Effects of stress on behavior and resting-state fMRI in rats and evaluation of Telmisartan therapy in a stress-induced depression model. BMC Psychiatry 18:337. 10.1186/s12888-018-1880-y 30333002 PMC6192217

[B46] Liu L, Zhou X, Zhang Y, Liu Y, Yang L, Pu J, Zhu D, Zhou C, Xie P (2016) The identification of metabolic disturbances in the prefrontal cortex of the chronic restraint stress rat model of depression. Behav Brain Res 305:148–156. 10.1016/j.bbr.2016.03.005 26947756

[B47] Lu H, Zou Q, Gu H, Raichle ME, Stein EA, Yang Y (2012) Rat brains also have a default mode network. Proc Natl Acad Sci USA 109:3979–3984. 10.1073/pnas.1200506109 22355129 PMC3309754

[B48] Luykx JJ, Laban KG, van den Heuvel MP, Boks MPM, Mandl RCW, Kahn RS, Bakker SC (2012) Region and state specific glutamate downregulation in major depressive disorder: a meta-analysis of (1)H-MRS findings. Neurosci Biobehav Rev 36:198–205. 10.1016/j.neubiorev.2011.05.014 21672551

[B49] Magalhães R, Barrière DA, Novais A, Marques F, Marques P, Cerqueira J, Sousa JC, Cachia A, Boumezbeur F, Bottlaender M, Jay TM, Mériaux S, Sousa N (2018) The dynamics of stress: a longitudinal MRI study of rat brain structure and connectome. Mol Psychiatry 23:1998–2006. 10.1038/mp.2017.244 29203852

[B50] Magalhães R, Novais A, Barrière DA, Marques P, Marques F, Sousa JC, Cerqueira JJ, Cachia A, Jay TM, Bottlaender M, Sousa N, Mériaux S, Boumezbeur F (2019) A resting-state functional MR imaging and spectroscopy study of the dorsal hippocampus in the chronic unpredictable stress rat model. J Neurosci 39:3640–3650. 10.1523/JNEUROSCI.2192-18.2019 30804096 PMC6510342

[B51] Manoliu A, Meng C, Brandl F, Doll A, Tahmasian M, Scherr M, Schwerthöffer D, Zimmer C, Förstl H, Bäuml J, Riedl V, Wohlschläger AM, Sorg C (2014) Insular dysfunction within the salience network is associated with severity of symptoms and aberrant inter-network connectivity in major depressive disorder. Front Hum Neurosci 7:930. 10.3389/fnhum.2013.00930 24478665 PMC3896989

[B52] Marrocco J, Reynaert M-L, Gatta E, Gabriel C, Mocaër E, Di Prisco S, Merega E, Pittaluga A, Nicoletti F, Maccari S, Morley-Fletcher S, Mairesse J (2014) The effects of antidepressant treatment in prenatally stressed rats support the glutamatergic hypothesis of stress-related disorders. J Neurosci 34:2015–2024. 10.1523/JNEUROSCI.4131-13.2014 24501344 PMC6608531

[B53] McKinnon MC, Yucel K, Nazarov A, MacQueen GM (2009) A meta-analysis examining clinical predictors of hippocampal volume in patients with major depressive disorder. J Psychiatry Neurosci 34:41–54. 19125212 PMC2612082

[B54] Mengler L, Khmelinskii A, Diedenhofen M, Po C, Staring M, Lelieveldt BPF, Hoehn M (2014) Brain maturation of the adolescent rat cortex and striatum: changes in volume and myelination. Neuroimage 84:35–44. 10.1016/j.neuroimage.2013.08.034 23994458

[B55] Mirza Y, Tang J, Russell A, Banerjee SP, Bhandari R, Ivey J, Rose M, Moore GJ, Rosenberg DR (2004) Reduced anterior cingulate cortex glutamatergic concentrations in childhood major depression. J Am Acad Child Adolesc Psychiatry 43:341–348. 10.1097/00004583-200403000-00017 15076268

[B56] Moriguchi S, Takamiya A, Noda Y, Horita N, Wada M, Tsugawa S, Plitman E, Sano Y, Tarumi R, ElSalhy M, Katayama N, Ogyu K, Miyazaki T, Kishimoto T, Graff-Guerrero A, Meyer JH, Blumberger DM, Daskalakis ZJ, Mimura M, Nakajima S (2019) Glutamatergic neurometabolite levels in major depressive disorder: a systematic review and meta-analysis of proton magnetic resonance spectroscopy studies. Mol Psychiatry 24:952–964. 10.1038/s41380-018-0252-9 30315224 PMC6755980

[B57] Mulders PC, van Eijndhoven PF, Schene AH, Beckmann CF, Tendolkar I (2015) Resting-state functional connectivity in major depressive disorder: a review. Neurosci Biobehav Rev 56:330–344. 10.1016/j.neubiorev.2015.07.014 26234819

[B58] Paasonen J, Stenroos P, Salo RA, Kiviniemi V, Gröhn O (2018) Functional connectivity under six anesthesia protocols and the awake condition in rat brain. Neuroimage 172:9–20. 10.1016/j.neuroimage.2018.01.014 29414498

[B59] Papp EA, Leergaard TB, Calabrese E, Johnson GA, Bjaalie JG (2014) Waxholm Space atlas of the Sprague Dawley rat brain. Neuroimage 97:374–386. 10.1016/j.neuroimage.2014.04.001 24726336 PMC4160085

[B60] Paulus MP, Stein MB (2010) Interoception in anxiety and depression. Brain Struct Funct 214:451–463. 10.1007/s00429-010-0258-9 20490545 PMC2886901

[B61] Pfleiderer B, Michael N, Erfurth A, Ohrmann P, Hohmann U, Wolgast M, Fiebich M, Arolt V, Heindel W (2003) Effective electroconvulsive therapy reverses glutamate/glutamine deficit in the left anterior cingulum of unipolar depressed patients. Psychiat Res Neuroim 122:185–192. 10.1016/S0925-4927(03)00003-9 12694892

[B62] Pineda JA (2008) Sensorimotor cortex as a critical component of an 'extended' mirror neuron system: does it solve the development, correspondence, and control problems in mirroring? Behav Brain Funct 4:47. 10.1186/1744-9081-4-47 18928566 PMC2577683

[B63] Portella MJ, de Diego-Adeliño J, Gómez-Ansón B, Morgan-Ferrando R, Vives Y, Puigdemont D, Pérez-Egea R, Ruscalleda J, Enric Á, Pérez V (2011) Ventromedial prefrontal spectroscopic abnormalities over the course of depression: a comparison among first episode, remitted recurrent and chronic patients. J Psychiatr Res 45:427–434. 10.1016/j.jpsychires.2010.08.010 20875647

[B64] Provencher SW (2001) Automatic quantitation of localized in vivo 1H spectra with LCModel. NMR Biomed 14:260–264. 10.1002/nbm.698 11410943

[B65] Rao U, Chen L-A, Bidesi AS, Shad MU, Thomas MA, Hammen CL (2010) Hippocampal changes associated with early-life adversity and vulnerability to depression. Biol Psychiatry 67:357–364. 10.1016/j.biopsych.2009.10.017 20015483 PMC2821020

[B66] Rocher C, Spedding M, Munoz C, Jay TM (2004) Acute stress-induced changes in hippocampal/prefrontal circuits in rats: effects of antidepressants. Cereb Cortex 14:224–229. 10.1093/cercor/bhg122 14704220

[B67] Rolls ET, Cheng W, Gong W, Qiu J, Zhou C, Zhang J, Lv W, Ruan H, Wei D, Cheng K, Meng J, Xie P, Feng J (2019) Functional connectivity of the anterior cingulate cortex in depression and in health. Cereb Cortex 29:3617–3630. 10.1093/cercor/bhy236 30418547

[B68] Rorden C, Karnath H-O, Bonilha L (2007) Improving lesion-symptom mapping. J Cogn Neurosci 19:1081–1088. 10.1162/jocn.2007.19.7.1081 17583985

[B69] Sanacora G, Treccani G, Popoli M (2012) Towards a glutamate hypothesis of depression: an emerging frontier of neuropsychopharmacology for mood disorders. Neuropharmacology 62:63–77. 10.1016/j.neuropharm.2011.07.036 21827775 PMC3205453

[B70] Seewoo BJ, Feindel KW, Etherington SJ, Rodger J (2018) Resting-state fMRI study of brain activation using low-intensity repetitive transcranial magnetic stimulation in rats. Sci Rep 8:6706. 10.1038/s41598-018-24951-6 29712947 PMC5928106

[B71] Seewoo BJ, Feindel KW, Etherington SJ, Rodger J (2019) Frequency-specific effects of low-intensity rTMS can persist for up to 2 weeks post-stimulation: a longitudinal rs-fMRI/MRS study in rats. Brain Stimul 12:1526–1536. 10.1016/j.brs.2019.06.028 31296402

[B72] Seewoo BJ, Joos AC, Feindel KW (2020) An analytical workflow for seed-based correlation and independent component analysis in interventional resting-state fMRI studies. Neurosci Res, in press. 10.1016/j.neures.2020.05.006 32464181

[B73] Sergejeva M, Papp EA, Bakker R, Gaudnek MA, Okamura-Oho Y, Boline J, Bjaalie JG, Hess A (2015) Anatomical landmarks for registration of experimental image data to volumetric rodent brain atlasing templates. J Neurosci Methods 240:161–169. 10.1016/j.jneumeth.2014.11.005 25445058

[B74] Shao Y, Yan G, Xuan Y, Peng H, Huang Q-J, Wu R, Xu H (2015) Chronic social isolation decreases glutamate and glutamine levels and induces oxidative stress in the rat hippocampus. Behav Brain Res 282:201–208. 10.1016/j.bbr.2015.01.005 25591473

[B75] Sheline YI (2011) Depression and the hippocampus: cause or effect? Biol Psychiatry 70:308–309. 10.1016/j.biopsych.2011.06.006 21791257 PMC3733566

[B76] Sheline YI, Barch DM, Price JL, Rundle MM, Vaishnavi SN, Snyder AZ, Mintun MA, Wang S, Coalson RS, Raichle ME (2009) The default mode network and self-referential processes in depression. Proc Natl Acad Sci USA 106:1942–1947. 10.1073/pnas.0812686106 19171889 PMC2631078

[B77] Slattery DA, Cryan JF (2012) Using the rat forced swim test to assess antidepressant-like activity in rodents. Nat Protoc 7:1009–1014. 10.1038/nprot.2012.044 22555240

[B78] Stepanichev M, Dygalo NN, Grigoryan G, Shishkina GT, Gulyaeva N (2014) Rodent models of depression: neurotrophic and neuroinflammatory biomarkers. Biomed Res Int 2014:932757. 10.1155/2014/932757 24999483 PMC4066721

[B79] Suvrathan A, Tomar A, Chattarji S (2010) Effects of chronic and acute stress on rat behaviour in the forced-swim test. Stress 13:533–540. 10.3109/10253890.2010.489978 20666651

[B80] Tambalo S, Peruzzotti-Jametti L, Rigolio R, Fiorini S, Bontempi P, Mallucci G, Balzarotti B, Marmiroli P, Sbarbati A, Cavaletti G, Pluchino S, Marzola P (2015) Functional magnetic resonance imaging of rats with experimental autoimmune encephalomyelitis reveals brain cortex remodeling. J Neurosci 35:10088–10100. 10.1523/JNEUROSCI.0540-15.2015 26157006 PMC4495237

[B81] Ulloa JL, Castañeda P, Berríos C, Díaz-Veliz G, Mora S, Bravo JA, Araneda K, Menares C, Morales P, Fiedler JL (2010) Comparison of the antidepressant sertraline on differential depression-like behaviors elicited by restraint stress and repeated corticosterone administration. Pharmacol Biochem Behav 97:213–221. 10.1016/j.pbb.2010.08.001 20705085

[B82] Videbech P, Ravnkilde B (2004) Hippocampal volume and depression: a meta-analysis of MRI studies. Am J Psychiatry 161:1957–1966. 10.1176/appi.ajp.161.11.1957 15514393

[B83] Walf AA, Frye CA (2007) The use of the elevated plus maze as an assay of anxiety-related behavior in rodents. Nat Protoc 2:322–328. 10.1038/nprot.2007.44 17406592 PMC3623971

[B84] Walter M, Henning A, Grimm S, Schulte RF, Beck J, Dydak U, Schnepf B, Boeker H, Boesiger P, Northoff G (2009) The relationship between aberrant neuronal activation in the pregenual anterior cingulate, altered glutamatergic metabolism, and anhedonia in major depression. Arch Gen Psychiatry 66:478–486. 10.1001/archgenpsychiatry.2009.39 19414707

[B85] Wang L, Hermens DF, Hickie IB, Lagopoulos J (2012) A systematic review of resting-state functional-MRI studies in major depression. J Affect Disord 142:6–12. 10.1016/j.jad.2012.04.013 22858266

[B86] Wang Q, Timberlake MA 2nd, Prall K, Dwivedi Y (2017) The recent progress in animal models of depression. Prog Neuropsychopharmacol Biol Psychiatry 77:99–109. 10.1016/j.pnpbp.2017.04.008 28396255 PMC5605906

[B87] Welniak–Kaminska M, Fiedorowicz M, Orzel J, Bogorodzki P, Modlinska K, Stryjek R, Chrzanowska A, Pisula W, Grieb P (2019) Volumes of brain structures in captive wild-type and laboratory rats: 7T magnetic resonance in vivo automatic atlas-based study. PLoS One 14:e0215348. 10.1371/journal.pone.0215348 30973956 PMC6459519

[B88] Willner P, Towell A, Sampson D, Sophokleous S, Muscat R (1987) Reduction of sucrose preference by chronic unpredictable mild stress, and its restoration by a tricyclic antidepressant. Psychopharmacology (Berl) 93:358–364. 10.1007/BF00187257 3124165

[B89] Wood T (2018) QUIT: quantitative imaging tools. J Open Source Softw 3:656.

[B90] Woolrich M (2008) Robust group analysis using outlier inference. Neuroimage 41:286–301. 10.1016/j.neuroimage.2008.02.042 18407525

[B91] Woolrich MW, Behrens TEJ, Beckmann CF, Jenkinson M, Smith SM (2004) Multilevel linear modelling for FMRI group analysis using Bayesian inference. Neuroimage 21:1732–1747. 10.1016/j.neuroimage.2003.12.023 15050594

[B92] Worsley KJ (2001) Statistical analysis of activation images In: Functional MRI: an introduction to methods (Jezzard P, Matthews PM, Smith SM, eds), pp 251–270. Oxford: Oxford University Press.

[B93] Xu J, Dydak U, Harezlak J, Nixon J, Dzemidzic M, Gunn AD, Karne HS, Anand A (2013a) Neurochemical abnormalities in unmedicated bipolar depression and mania: a 2D 1H MRS investigation. Psychiatry Res 213:235–241. 10.1016/j.pscychresns.2013.02.008 23810639 PMC3729606

[B94] Xu S, Ji Y, Chen X, Yang Y, Gullapalli RP, Masri R (2013b) In vivo high-resolution localized (1) H MR spectroscopy in the awake rat brain at 7 T. Magn Reson Med 69:937–943. 10.1002/mrm.24321 22570299 PMC3427395

[B95] Yildiz-Yesiloglu A, Ankerst DP (2006) Review of 1H magnetic resonance spectroscopy findings in major depressive disorder: a meta-analysis. Psychiatry Res 147:1–25. 10.1016/j.pscychresns.2005.12.004 16806850

[B96] Yin Z, Chang M, Wei S, Jiang X, Zhou Y, Cui L, Lv J, Wang F, Tang Y (2018) Decreased functional connectivity in insular subregions in depressive episodes of bipolar disorder and major depressive disorder. Front Neurosci 12:842. 10.3389/fnins.2018.00842 PMC6246657 30487732

[B97] Zarrinpar A, Deldin P, Kosslyn SM (2006) Effects of depression on sensory/motor vs. central processing in visual mental imagery. Cogn Emot 20:737–758. 10.1080/02699930500405600

[B98] Zheng C, Quan M, Zhang T (2012) Decreased thalamo-cortical connectivity by alteration of neural information flow in theta oscillation in depression-model rats. J Comput Neurosci 33:547–558. 10.1007/s10827-012-0400-1 22648379

[B99] Zhu X, Wang X, Xiao J, Liao J, Zhong M, Wang W, Yao S (2012) Evidence of a dissociation pattern in resting-state default mode network connectivity in first-episode, treatment-naive major depression patients. Biol Psychiatry 71:611–617. 10.1016/j.biopsych.2011.10.035 22177602

